# Discovering Interpretable Semantics from Radio Signals for Contactless Cardiac Monitoring

**DOI:** 10.1002/advs.202524283

**Published:** 2026-03-15

**Authors:** Jinbo Chen, Haoyu Wang, Guixin Xu, Yuqin Yuan, Xinmeng Cai, Zhengwu Wei, Dongheng Zhang, Qibin Sun, Tianwei Zhang, Cuntai Guan, Yan Chen

**Affiliations:** ^1^ School of Cyber Science and Technology University of Science and Technology of China Hefei China; ^2^ College of Computing and Data Science Nanyang Technological University Singapore Singapore; ^3^ Zhongke Radio Sensing AI Technology Hefei China

**Keywords:** cardiac monitoring, radio signal, semantic representation

## Abstract

Radio signals have emerged as a promising modality for cardiac monitoring, enabling fully contactless and operation‐free measurement. However, the lack of semantic understanding, i.e., the ability to interpret signal dynamics in clinically meaningful terms, remains a fundamental barrier to performance, interpretability, and clinical translation. Here, we introduce a semantic representation framework for radio‐based cardiac monitoring, grounded in an information bottleneck formulation. Our approach leverages intrinsic semantic invariance in the signal by integrating intra‐modal variability compression with cross‐modal semantic alignment. This enables the transformation of radio measurements into a structured representation space where cardiac semantics are encoded in an interpretable and clinically meaningful manner. We validate the proposed framework on a large‐scale cohort of 9518 outpatients. The learned semantic representations exhibit strong alignment with reference semantics. Building on these representations, our method achieves interpretable and clinical‐grade cardiac monitoring, including heart rhythm monitoring with a median inter‐beat interval error of 9.4 ms (95% CI: 9.3–9.6), and arrhythmia diagnosis for atrial fibrillation and premature beats, with F1 scores of 0.929 (95% CI: 0.906–0.948) and 0.867 (95% CI: 0.847–0.887), respectively. We also demonstrate the effectiveness of the proposed framework in practical long‐term, daily‐life deployment scenarios. These results highlight semantic representation as a key enabler for achieving reliable and transparent radio cardiac monitoring.

## Graphical abstract

This study presents a semantic representation framework for clinically interpretable cardiac monitoring from contactless radio signals. It formulates radio semantic learning as an information‐bottleneck problem and approximates the objective via intra‐modal compression and cross‐modal alignment, structuring radio measurements into meaningful semantic factors. Validated on 9518 outpatients, it achieves clinical‐grade heart rhythm monitoring and arrhythmia diagnosis with transparent interpretability and robust real‐world deployment.

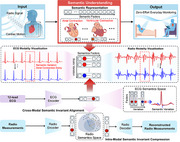

## 1 Introduction

Human recognition fundamentally relies on semantic understanding [1, 2], the interpretable meanings behind observations. In perceptual modalities like vision and auditory processing, semantics provide the foundation for moving beyond raw signals toward meaningful interpretation and reasoning [3, 4, 5, 6, 7]. Similarly, cardiac monitoring is grounded in semantic frameworks that map signal patterns to “semantics,” which refers to the physiological meaning of cardiac dynamics underlying the measured signals  [8, 9, 10], built upon long‐standing clinical knowledge and empirical validation. Recently, the use of radio signals for cardiac monitoring has attracted significant attention  [11, 12, 13, 14, 15, 16, 17, 18], as it enables fully contactless and operation‐free measurement. This overcomes key limitations of current contact‐based methods, which are often uncomfortable and inconvenient for long‐term use  [19, 20]. This shift paves the way for transitioning from hospital‐centered passive monitoring to personalized, proactive care via zero‐effort, continuous daily‐life monitoring. However, as an emerging modality, the semantic understanding of radio measurements still remains unexplored. This semantic gap limits both monitoring performance and interpretability, ultimately hindering the clinical translation of this technology.

The mechanism underlying radio cardiac monitoring relies on the ability of radio signals to capture cardiac motions  [21]. As the heart beats, its mechanical activity propagates through internal tissues to the torso surface, generating subtle displacements. These motions modulate the phase of reflected radio signals, enabling the extraction of detailed heartbeat information through signal analysis  [16].

**FIGURE 1:**
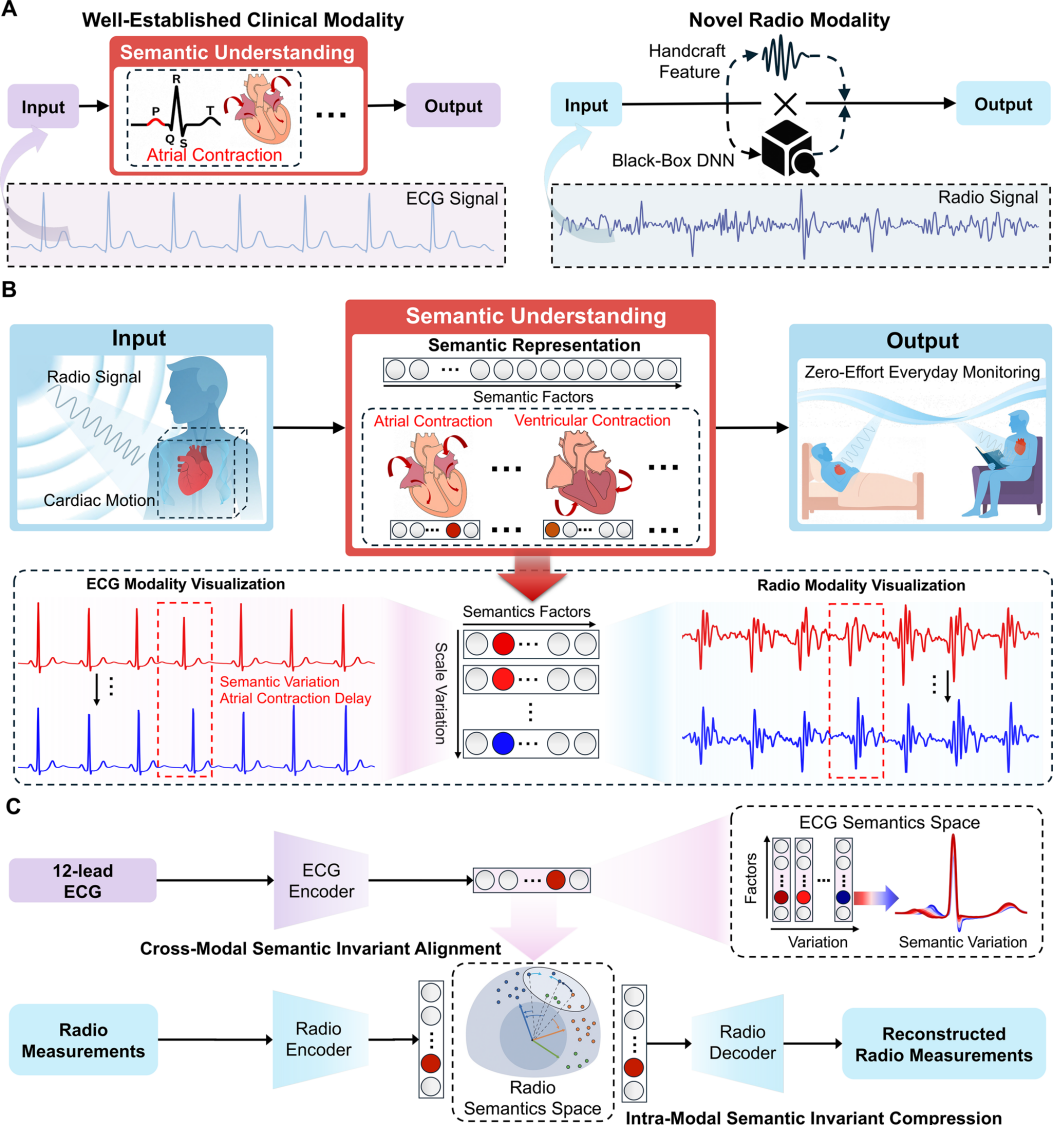
System Overview. (A) In well‐established clinical ECG monitoring, signal interpretation is grounded in the semantic understanding of the PQRST complex. In contrast, existing approaches for the emerging radio modality all bypass this semantic layer, relying instead on handcrafted feature extraction or end‐to‐end black‐box model. (B) Our method transforms radio measurements into a representation space where cardiac semantics are encoded as factors variation. The representation can be further visualized across both radio and ECG modalities, revealing how cardiac states are expressed in signal forms. This semantic layer provides a reliable foundation for radio cardiac monitoring, fully unlocking its advantages for zero‐effort, everyday monitoring. (C) Our semantic modeling is designed by leveraging semantic invariance both within and across modalities. Intra‐modal invariance is enforced through signal compression across semantically invariant signal transformations. Cross‐modal invariance is achieved via an ECG semantic alignment. The ECG semantic space is pretrained on a large‐scale ECG‐only dataset. After pretraining, the resulting ECG semantic representations capture meaningful variations in cardiac dynamics, serving as a semantic reference for grounding radio semantics modeling.

While the principle is straightforward, its clinical applicability remains limited by the absence of semantic representations that align signal dynamics with clinical understanding, rather than providing superficial labels. In well‐established biosignals such as the electrocardiogram (ECG), decades of clinical experience have led to the development of effective semantic frameworks. In ECG, semantic representation is grounded in the morphological characteristics of the PQRST complex, with each component corresponding to physiological functions of the heart  [8, 22], as shown in Figure 1. However, this process is labor‐intensive and inherently slow to scale, especially for emerging sensing modalities where clinical priors and interpretive conventions are yet to be established. Consequently, existing methods all bypass the semantic layer, relying instead on either handcrafted signal‐level features  [11, 13, 15, 23, 24], or implicit black‐box inference, such as end‐to‐end models  [12, 14, 17] or task‐specific feature distillation  [18], as shown in Figure 1A. These approaches lack the transparency and generalizability that are crucial in medical applications and have become a growing concern in recent years  [25, 26].

To address this gap, we introduce a novel semantic representation modeling framework for the emerging radio modality, moving beyond traditional empirical semantic analysis toward scalable, data‐driven discovery. Specifically, we propose to formulate semantic modeling as an information bottleneck problem and leverage intrinsic semantic invariance within the signal to approximate its solution directly from data. These semantic invariances are twofold. First, across signal transformations during contactless measurement, the underlying physiological semantics remain unchanged. We leverage this intra‐modal semantic invariance as the foundation for semantic compression. Second, the inherent coupling between cardiac electrical and mechanical activities  [27] ensures a shared semantic basis across ECG and radio modalities, enabling cross‐modal alignment for semantic grounding. By compressing intra‐modal variability and aligning with cross‐modal invariability, we construct a representation space in which semantics are encoded as interpretable and meaningful structures, enabling the transformation of contactless radio measurements into interpretable semantic representations, as shown in Figure 1B.

We evaluate the proposed framework on a large‐scale cohort of 9518 outpatients across multiple dimensions, ranging from the fidelity of the encoded semantics to its effectiveness in supporting clinical monitoring. First, the learned semantic representations show strong alignment with semantic references, with an average correlation of R = 0.885 (P<0.001) across three quantifiable semantic groundtruth. Second, based on these representations, the framework enables clinical‐grade cardiac monitoring with transparent interpretability. For arrhythmia diagnosis, it achieves F1 scores of 0.929 (95% CI: 0.906–0.948) and 0.867 (95% CI: 0.847–0.887) for atrial fibrillation and premature beats, respectively, approaching the performance of ECG‐model‐based methods. For cardiac rhythm monitoring, it improves precision by more than tenfold compared with state‐of‐the‐art (SOTA) radio‐based approaches, yielding a median inter‐beat‐interval error of 9.4 ms (95% CI: 9.3–9.6). Moreover, we demonstrate the practical robustness and efficiency of the proposed method under long‐term daily‐life deployment, validated over 48 subjects and 276 h of continuous monitoring. We believe these results lay a solid foundation for the clinical translation of radio cardiac monitoring, where semantic modeling ensures both reliable performance and transparent clinical interpretation.

## 2 Semantic Modeling of Radio Signals

Semantic modeling aims to identify and represent the meaningful components embedded in raw observations. In the context of radio cardiac monitoring, the raw observations are body reflection signals, where cardiac motion is modulated as phase variations. We model this process as an information bottleneck problem  [28, 29]: given radio measurements $X_{R}$, the objective is to find latent representations $Z_{R}$ that retain maximal mutual information with the desired cardiac semantics S, while minimizing mutual information with the radio signal itself $X_{R}$:

(1) $$\min_{p(Z_{R}|X_{R})}I\left(Z_{R};X_{R}\right)-\beta I\left(Z_{R};S\right)$$

where β is a Lagrange multiplier that controls the trade‐off between compression and semantic preservation. However, directly solving this objective is non‐trivial in the context of radio signals: the term $I(Z_{R};S)$ cannot be computed because annotations of the target semantics S are unavailable, and the term $I(Z_{R};X_{R})$ is difficult to characterize because the signal's semantic components are weak relative to the complex variations introduced during contactless measurement. To address the above challenges, we propose exploiting the intrinsic semantic invariance within the signal to solve the problem in a data‐driven manner. As shown in Figure 1C, our approach incorporates two core designs: we leverage intra‐modal semantic invariant compression to regularize $I(Z_{R};X_{R})$, and we utilize cross‐modal semantic invariant alignment to approximate $I(Z_{R};S)$.

### 2.1 Intra‐Modal Semantic Invariant Compression

Radio signal measurements are noisy and high‐dimensional, but the semantic information they convey is inherently low‐dimensional, aligning with the objective of minimizing $I(Z_{R};X_{R})$. We implement this using a self‐supervised compression–reconstruction framework, where a neural encoder compresses the radio measurements into a compact latent representation, and a decoder reconstructs the original input. To ensure that the latent space captures physiological semantics rather than dominant waveform variations driven by signal propagation during measurement, we design semantic‐invariant signal transformations and enforce consistency of the latent representations across these transformations. These transformations are derived from the underlying mechanisms of signal propagation, effectively constraining the latent space to preserve semantics while being invariant to nuisance variations.

### 2.2 Cross‐Modal Semantic Invariant Alignment

The cardiac excitation–contraction coupling mechanism  [27] reveals a nonlinear relationship between electrical activity and mechanical motion in the heart  [30]. This establishes a cross‐modal semantic bridge between the mechanical motion captured by radio signals and the electrical activity recorded in the ECG, implying that synchronized ECG and radio measurements encode the same underlying cardiac semantics. Motivated by this insight, we propose to regulate the semantic modeling by aligning the learned radio representations with the semantic reference from the ECG modality, thereby efficiently approximating $I(Z_{R};S)$. Specifically, we first construct an ECG semantic space using a variational autoencoder (VAE)  [31] pretrained on a large‐scale ECG‐only dataset. VAEs have been shown to effectively learn semantic representations across modalities  [3, 5, 32, 33]. In particular, for ECG signals, prior work demonstrates that such representations can capture the semantics of the PQRST complex, enabling interpretable downstream analysis  [22]. We then use synchronized recordings of radio and ECG signals to extract paired representations and impose cross‐modal semantic alignment by encouraging the radio embeddings to match their ECG counterparts in the reference semantic space.

The above design yields a semantic representation of radio signals. The interpretation of the learned factors is provided at two levels: (i) direct factor‐to‐semantic readouts, where semantic quantities can be obtained by using a linear mapping optimized with annotation, and (ii) factor‐to‐ECG visualization, where the factors can be decoded into clinically familiar ECG waveforms (and corresponding radio‐domain reconstructions) via modality‐specific decoders within our framework. This bi‐directional visualization capability provides direct insight into cardiac states, bridging physiological meaning and signal expression. A detailed description of the modeling architecture is provided in the Methods section.

## 3 Results

### 3.1 Radio Semantic Modeling Evaluation

**FIGURE 2:**
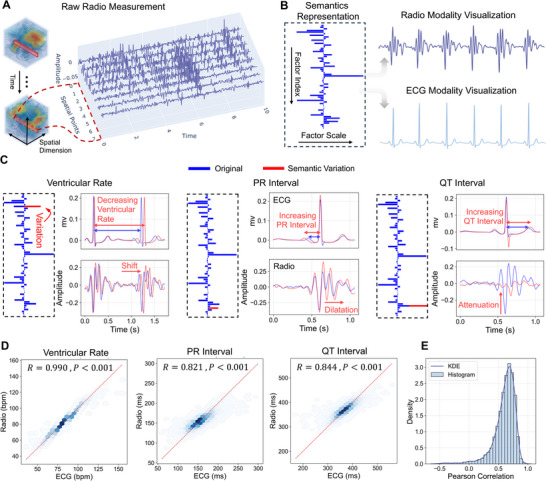
Radio semantics modeling results. (A) Raw radio measurements are inherently high‐dimensional and lack semantic interpretability. (B) Our method transforms raw measurements into 64 semantic factors to represent the underlying cardiac semanctics, which can be further visualized in both radio and ECG modalities for interpretability. (C) Demonstration of radio semantic factors representing cardiac semantics and corresponding signal expressions in both radio and ECG modalities. In each semantic subplot, the left panel shows the semantic factors, the top‐right panel shows the radio waveform visualization, and the bottom‐right panel shows the ECG waveform visualization. Blue traces correspond to the original semantic state, while red traces represent the changes induced by varying the associated semantic factor, illustrating the resulting semantic variation in the visualized signals. (D) Hexagon plot illustrating the alignment between the radio semantic factors and the semantic groundtruth (n = 9518 subjects). (E) Distribution of correlation coefficients between semantic spaces of radio and ECG modalities.

We first compare the original radio measurements with the output of our semantic modeling. As shown in Figure 2A, the high‐dimensional spatiotemporal radio measurements, which capture the full cardiac mechanical motion across the body, lack explicit semantic structure and are intrinsically uninterpretable. Our method transforms these raw measurements into 64 semantic factors representing the underlying cardiac dynamics (Figure 2B). These factors can be visualized in both the radio waveform and the clinically familiar ECG waveform, enabling cross‐modal interpretation.

To demonstrate the semantic expressiveness of the learned factors, we first show how the radio‐derived semantic factors reflect cardiac semantics and how their variations influence corresponding signal expressions across both modalities. Specifically, we select ventricular rate, PR interval, and QT interval as semantic references  [34]. These metrics respectively characterize the overall duration of the cardiac cycle, the temporal coordination between atrial and ventricular contractions, and the duration of a complete ventricular contraction–relaxation process, forming the foundation of ECG interpretation and widely used in clinical diagnosis  [8]. For each semantic reference, we identify the top‐correlated radio semantic factor and vary its scale to visualize how it drives signal patterns within a single cardiac cycle across both modalities, as shown in Figure 2C. Across all semantics, the radio modality exhibits a heartbeat complex trend structure similar to that in ECG, dominated by a prominent peak similar to the R‐wave in ECG. Changes in ventricular rate are reflected in the ECG as a shift of the PQRST complex, and correspondingly, in the radio modality as a trend complex shift. For an increase in PR interval, the ECG shows an extended interval between the P‐R wave, while in the radio modality, this is primarily manifested as a dilation of the trend complex, indicating a prolonged coordination time of atrial and ventricular mechanical motion. For an increase in QT interval, the ECG exhibits an extended Q–T segment, whereas the radio modality shows an attenuation of the trend complex amplitude, implying that the mechanical energy is distributed over a longer contraction–relaxation cycle, leading to a reduced mechanical impulse.

A detailed demonstration of how the semantic factors extend from a single cardiac cycle and evolve over time during continuous monitoring is provided in the Note S1.1. To further quantify how accurately the learned representation supports semantic readouts, we learn a linear readout (linear regression) from the factor space to each semantic quantity using annotated training data, and evaluate it on held‐out subjects following the K‐fold cross‐validation strategy described in Section 6. We then report the Pearson correlation between the predicted semantic readouts and the groundtruth. As illustrated in the hexagon plot in Figure 2D, the learned representations exhibit strong correspondence with clinically established cardiac semantics, with R = 0.990 for ventricular rate, R = 0.821 for PR interval, and R = 0.844 for QT interval (all with P<0.001).

To evaluate the semantic fidelity from the feature perspective, we analyze the correlation between the constructed radio semantic space and the reference ECG semantic space. We project the semantic representations derived from synchronized radio and ECG measurements into a 2D UMAP manifold  [35] (Note S1.2), a technique widely used for visualizing high‐dimensional features. The visualization reveals a clear alignment trend between the two modalities in the semantic space. We then quantify this alignment by computing the correlation between each pair of synchronized radio and ECG representations. The distribution of correlation coefficients is shown in Figure 2E, yielding a median correlation of 0.647 (95% CI: 0.645–0.648).

### 3.2 Cardiac Rhythm Monitoring via Radio Semantics

Monitoring cardiac rhythm is the most fundamental downstream task in cardiac monitoring  [36]. Therefore, we evaluate the effectiveness of the proposed semantic representation in supporting this task. Cardiac rhythm is typically quantified by heart rate variability (HRV), which is derived from interbeat intervals (IBI) and summarized using standard statistical metrics, including the root mean square of successive differences (RMSSD), the standard deviation of normal‐to‐normal intervals (SDNN), and the proportion of successive intervals differing by more than 50 ms (pNN50) [36]. Clinically, IBI is obtained from ECG signals by identifying R‐peaks, which serve as well‐established semantic markers of individual cardiac cycles [37]. Following this pipeline, we extract HRV from the semantic ECG visualization by identifying heartbeats with R‐peak. We compare our approach with the current SOTA method that relies on signal modeling and detects heartbeat cycles based on handcrafted harmonic frequency patterns  [15].

**FIGURE 3:**
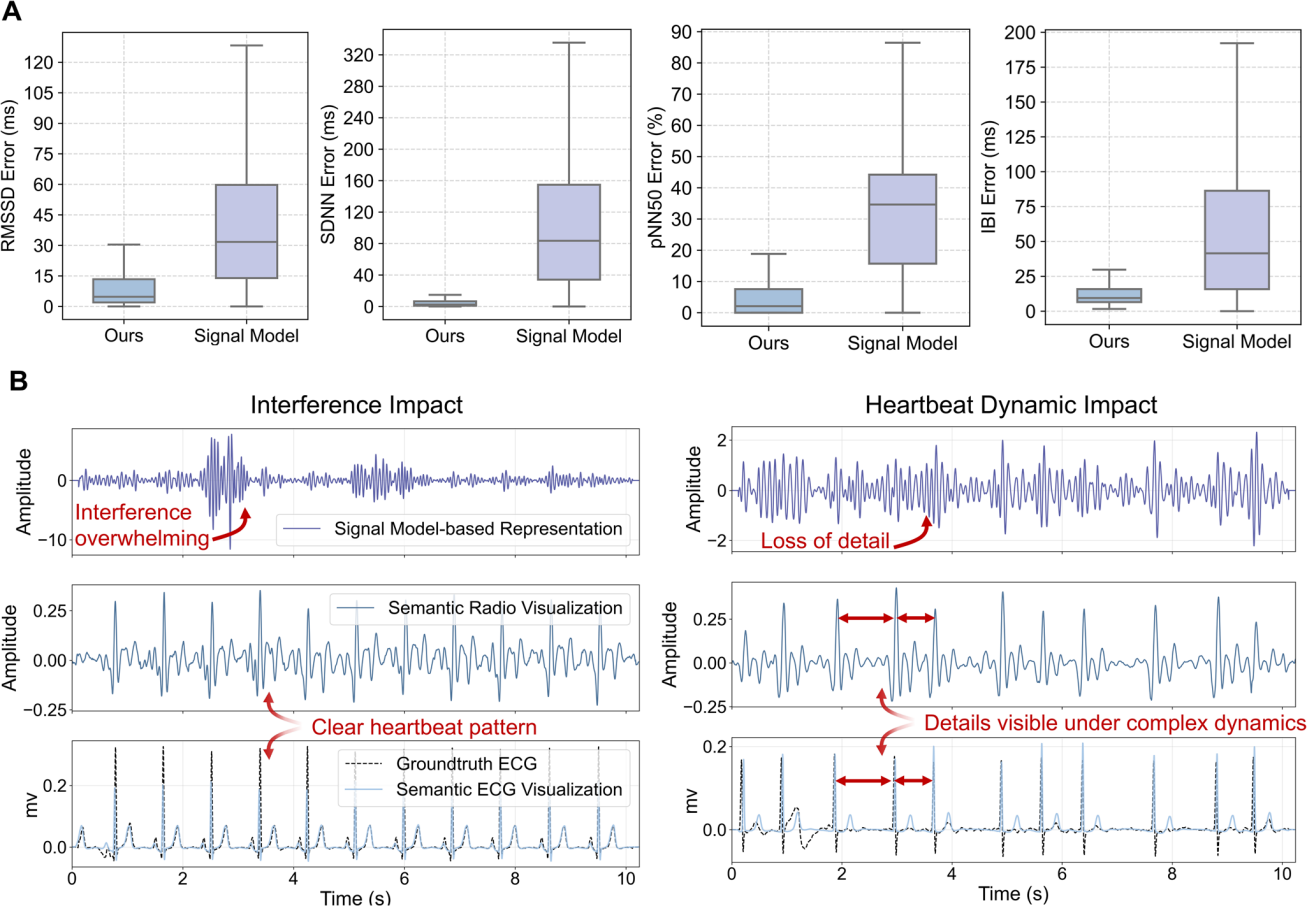
Cardiac rhythm monitoring results. (A) Comparison of error distributions in four HRV metrics of IBI, RMSSD, SDNN, and pNN50. (n = 9,518 subjects). Boxes show the median and interquartile range; whiskers indicate 1.5 times the IQR. (B) Comparison of our semantic visualizations with the groundtruth ECG and the signal‐model‐based baseline under representative conditions with interference and complex heartbeat dynamics.

As shown in Figure 3A, our method significantly outperforms the model‐based baseline, achieving more than a 12‐fold average improvement. For RMSSD, our method yields a median error of just 4.7 ms (95% CI: 4.6–4.9) compared to 83.5 ms (95% CI: 81.3–85.9) for the baseline, suggesting improved sensitivity to subtle beat‐to‐beat variability. For SDNN, which captures overall rhythm variability over time, our semantic‐based approach achieves a median error 2.5 ms (95% CI: 2.4–2.6), while the baseline exceeds 31.7 ms (95% CI: 30.9–32.5), highlighting our method's robustness in tracking long‐term rhythm structure. For pNN50, an established marker of parasympathetic activity, our method maintains a median error of 2.1% (95% CI: 0.8%–2.5%), in contrast to the baseline's 34.6% (95% CI: 30.9%–32.5%), underscoring the reliability of semantic representations in detecting physiologically meaningful variations. These performance gains stem from precise cardiac cycle localization, with our method achieving a median IBI error of 9.4 ms (95% CI: 9.3–9.6), substantially lower than the baseline's 41.5 ms (95% CI: 40.4–42.4).

This significant performance improvement is powered by our semantic understanding of the signal. In contrast, the baseline method relies on signal modeling with constrained assumptions about the signal's frequency distribution. Such assumptions are difficult to satisfy in scenarios, where time‐varying interference and complex heartbeat dynamics are present. We compare two representative examples in Figure 3B to illustrate the difference. It can be observed that when interference is present, even in the normal rhythm case (left panel), the baseline method is overwhelmed by noise, resulting in severe masking of the heartbeat pattern and making heartbeat cycle identification infeasible. In the irregular cardiac rhythms of atrial fibrillation case (right panel), despite the absence of interference, the baseline method produce disordered frequency components, leading to distorted waveforms with detail loss. In contrast, in both cases, our semantic radio visualization remains robust and exhibits clear, repetitive rhythmic structures with distinct and identifiable main peaks. Meanwhile, our semantic ECG visualization accurately captures the underlying cardiac dynamics, with identifiable R‐peaks that align with those in the groundtruth ECG.

### 3.3 Semantics Driven Arrhythmia Diagnosis

Arrhythmia diagnosis serves as another important task in cardiac monitoring, where reliable and timely detection is crucial for CVDs management [38, 39]. To evaluate the effectiveness of our pipeline, we select two clinically significant arrhythmias, atrial fibrillation (AF) and premature beats (PB), as diagnosis benchmark. These are among the most common arrhythmias globally and are associated with a high risk of severe complications [39, 40, 41]. We verify the diagnosis performance based on our semantic representation without any task‐specific fine‐tuning and construct an interpretable diagnostic pipeline by modeling arrhythmia classification as a linear projection from learned semantic factors to disease categories. We compare our method against two black box DNN baselines: #1  [17] is an end‐to‐end model that directly classifies arrhythmias from signals; #2  [18] is a knowledge transfer–based approach that leverages the ECG domain to guide diagnosis‐related feature extraction from signals, achieving current SOTA performance.

**FIGURE 4:**
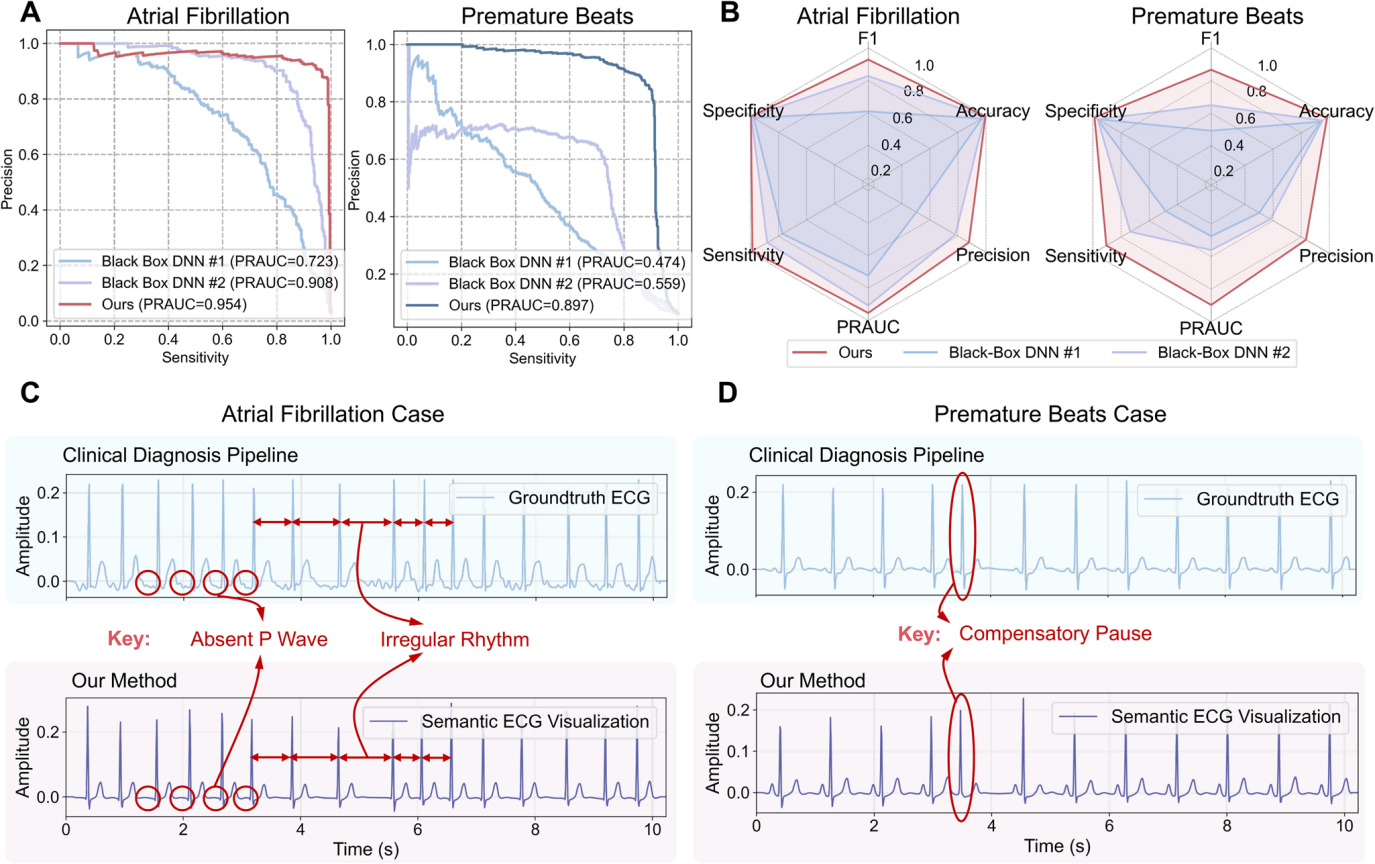
Semantic‐driven arrhythmia diagnosis results. (A) PRAUC comparison for atrial fibrillation (AF) and premature beats (PB) between our method and two black‐box baselines: #1  [17], #2  [18] (n = 9,518 subjects). (B) Radar plot summarizing key diagnosis metrics across AF and PB (n = 9,518 subjects). (C) Representative AF case visualized with semantic ECG and groundtruth ECG, showing that our method fully captures the key semantics used in the clinical diagnostic pipeline–irregular rhythm and absence of consistent P waves–consistent with the groundtruth ECG. (D) Representative PB case visualized with semantic ECG and groundtruth ECG, showing that our method fully captures the key semantics used in the clinical diagnostic pipeline–a compensatory pause–consistent with the groundtruth ECG.

As shown in Figure 4A, our method consistently outperforms both baselines with Precision–Recall Area Under the Curve (PRAUC) of 0.954 for AF and 0.897 for PB, compared to 0.723/0.474 (#1) and 0.908/0.559 (#2). Figure 4B further visualizes the diagnostic performance using radar plots, where our approach surpasses both baselines across all key metrics. For AF detection, while knowledge transfer yields better results than direct signal classification, our semantic method further boosts sensitivity from 0.880 (95% CI: 0.839–0.915) to 0.986 (95% CI: 0.970–0.997) and precision from 0.781 (95% CI: 0.737–0.826) to 0.878 (95% CI: 0.840–0.912), achieving an F1‐score of 0.929 (95% CI: 0.906–0.948). For PB detection, both baselines perform poorly, with F1‐scores of 0.493 (95% CI: 0.459–0.528) and 0.649 (95% CI: 0.618–0.677), respectively, failing to reliably capture the diagnostic patterns. In contrast, our method achieves an F1‐score of 0.867 (95% CI: 0.847–0.887). This large performance gap highlights the challenge of PB diagnosis, which requires fine‐grained semantic understanding like identifing atrioventricular timing that black‐box models fail to capture without structured semantic guidance.

To establish an upper‐bound reference, we further evaluated a gold‐standard ECG‐based method  [42] on our dataset. This method, known to exceed cardiologist‐level performance, achieved F1‐scores of 0.937 (95% CI: 0.916–0.956) for AF and 0.906 (95% CI: 0.890–0.922) for PB. In comparison, our framework approaches this benchmark, demonstrating that semantic representation enables clinical‐grade arrhythmia detection in this fully contactless modality.

We compare two representative cases using our semantic ECG visualization and the groundtruth ECG. As shown in Figure 4C, for the AF case, our method precisely captures the key semantics in the clinical diagnostic process, including the irregular R–R intervals and the absence of consistent P waves. As shown in Figure 4D, for the PB case, our method accurately captures the semantic feature of a compensatory pause following normal consecutive beats. Furthermore, our semantic‐driven diagnostic pipeline enables transparent decision‐making, as each diagnostic outcome can be directly traced to specific semantic factors. To further illustrate how the model utilizes the learned semantics for arrhythmia classification, we analyze its decision pathway in Note S1.3, where the results demonstrate decision logic of our semantic‐based linear diagnostic pipeline consistent with established clinical diagnostic reasoning. These findings indicate that our method effectively represents disease‐related semantics, providing a direct and interpretable pathway for reliable arrhythmia diagnosis.

### 3.4 Case Study: Exploring Contactless Cardiac Monitoring in Daily Life with Radio Semantics

Unlike short‐term clinical monitoring that provide only snapshot observations of cardiac status, long‐term daily‐life monitoring offers continuous and contextual insights into cardiovascular health. As an alternative to traditional monitoring tools, radio cardiac sensing is emerging to enable active, contactless, and zero‐effort monitoring in daily‐life. Therefore, such daily‐life validation serves as an important step toward demonstrating the translational impact of our semantic framework.

**FIGURE 5:**
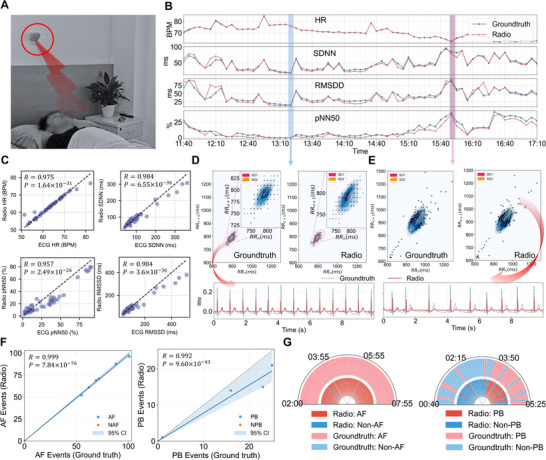
Long‐term monitoring results in daily‐life settings (A) Experimental setup for radio monitoring during overnight sleep. (B) HRV metric trends, including HR, SDNN, RMSSD, and pNN50, tracked over the sleep duration of a subject. Metrics are computed every 5 min. (C) Scatter plots comparing our method and groundtruth results for the four HRV metrics (n = 48 nights). (D) Poincaré plot over a 5‐min low‐variability segment during early sleep, alongside a short radio‐derived semantic ECG snippet from the same period (E) Poincaré plot over a 5‐min high‐variability segment during later sleep, alongside a short radio‐derived semantic ECG snippet from the same period. (F) Scatter plot comparing radio‐identified AF and PB events with groundtruth across overnight recordings (n = 48 nights). (G) Full‐night temporal dynamics for two representative subjects–one with AF (left) and one with PB (right). The semicircle represents the full‐night span.

To this end, we designed a daily‐life monitoring framework by seamlessly integrating our radio sensing system into participants' natural sleep environments, and conducted experiments to evaluate its effectiveness using the model trained in the preceding section, without any additional fine‐tuning. Implementation details of the experiments are provided in Note S2. Specifically, we recruited 48 subjects who reported discomfort and were scheduled for medical check‐ups. Before their hospital visits, we installed our radio monitoring device in each participant's bedroom and recorded overnight signals as they slept naturally (Figure 5A). Simultaneously, groundtruth ECG signals were collected using a Holter monitor. Throughout the monitoring process, participants maintained their usual sleep routines without any intervention, aside from the presence of a radar sensor mounted above the bed. After the recordings, certified cardiologists annotated the ECG data to serve as diagnostic reference for subsequent analysis.

We first evaluated the system's effectiveness in capturing HRV dynamics. As shown in Figure 5B, we show a representative subject results of HRV metrics including Heart Rate (HR), SDNN, RMSSD, and pNN50 dynamics over the sleep duration. The metrics are calculated every 5 min. It can be observed that our method accurately tracks the overnight trends of these metrics in agreement with groundtruth. Notably, during the early phase of sleep, the subject exhibits elevated HR and suppressed variability (lower SDNN, RMSSD, and pNN50), indicating reduced autonomic modulation. Later stages show a decline in HR and a concurrent increase in variability metrics, reflecting enhanced parasympathetic activity. In Figure 5D,E, we present Poincaré plots corresponding to two representative physiological states (each over a 5‐min window), along with the associated ECG waveform visualizations. During the early phase, the heartbeats exhibit low variability and cluster tightly around the center of the Poincaré plot. In contrast, during the later phase, the heartbeats appear more dispersed along the diagonal axis, and the semantic ECG visualization shows noticeable irregularities. Figure 5C summarizes the overnight HRV monitoring results across 48 subjects. Strong correlations were observed for HR (R=0.975, $P=1.64\times 10^{-31}$), pNN50 (R=0.957, $P=2.49\times 10^{-26}$), SDNN (R=0.984, $P=6.55\times 10^{-36}$), and RMSSD (R=0.984, $P=3.61\times 10^{-36}$), indicating high agreement with grondtruth.

We further evaluated the system's capability to detect arrhythmic events during overnight monitoring. Specifically, we assessed the identification of AF and PB episodes, comparing our results with groundtruths at a 5‐min temporal resolution. As illustrated in Figure 5F, all 6 subjects with AF and all 4 subjects with PB were successfully identified by our system. Moreover, the detected events closely matched the groundtruth throughout the night, demonstrating strong agreement with AF (R=0.999, $p=7.84\times 10^{-76}$) and PB (R=0.992, $p=9.60\times 10^{-43}$). Two representative overnight cases of AF and PB are shown in Figure 5G, depicting the full‐night temporal evolution of AF and PB episodes. Our system effectively tracked the temporal dynamics of these events in a fully passive and contactless manner.

### 3.5 Ablation Studies and Subanalyses

**FIGURE 6:**
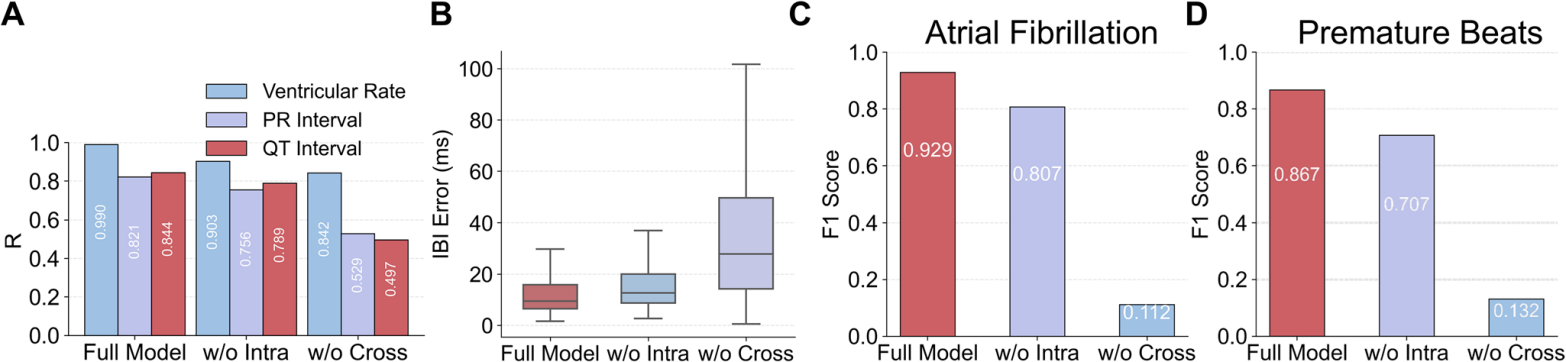
Ablation results of the two key modules: cross‐modal alignment and intra‐modal compression. The full model is compared with two ablated variants: w/o intra‐modal compression and w/o cross‐modal alignment. (A) Ablation results on factor‐to‐semantic readout performance. Numerical results of correlation (R) are provided in the plot (all p<0.001; n=9518). (B) Ablation results on downstream HRV monitoring performance. (all n=9518). (C) Ablation results on downstream AF diagnosis performance. (all n=9518). (D) Ablation results on downstream PB diagnosis performance.(all n=9518).

We first focus on the main component of the proposed method: cross‐modal alignment and intra‐modal compression. Specifically, we compare the full model with two ablated variants: w/o intra‐modal compression and w/o cross‐modal alignment. We evaluate these variants from the perspective of direct factor‐to‐semantic readout. As shown in Figure 6, cross‐modal alignment provides the primary contribution to semantic construction. Without this component, relying only on intra‐modal compression is insufficient to establish clinically meaningful semantics. The reason is that intra‐modal compression mainly encourages the model to compress the input into a compact representation, but it does not ensure that the compressed features are semantically meaningful. As a result, the learned representation may become more compact while still entangling heterogeneous signal components, and thus remains only weakly grounded in cardiac semantics. At the same time, the downstream‐task results further support this interpretation. As shown in Figure 6B–D, both HRV monitoring and AF/PB diagnosis indicate that cross‐modal alignment accounts for the majority of the performance gain, while removing it leads to a substantial degradation. Notably, intra‐modal compression provides a clear additional gain beyond cross‐modal alignment. Although cross‐modal alignment establishes the primary semantic correspondence, it does not fully remove modality‐specific redundancy and non‐semantic interference in radio signals. Intra‐modal compression further refines the latent representation by enforcing compactness and invariance, thereby improving semantic quality, robustness, and downstream performance. The above results indicate that both components contribute to the overall performance, while their roles are complementary rather than redundant.

**FIGURE 7:**
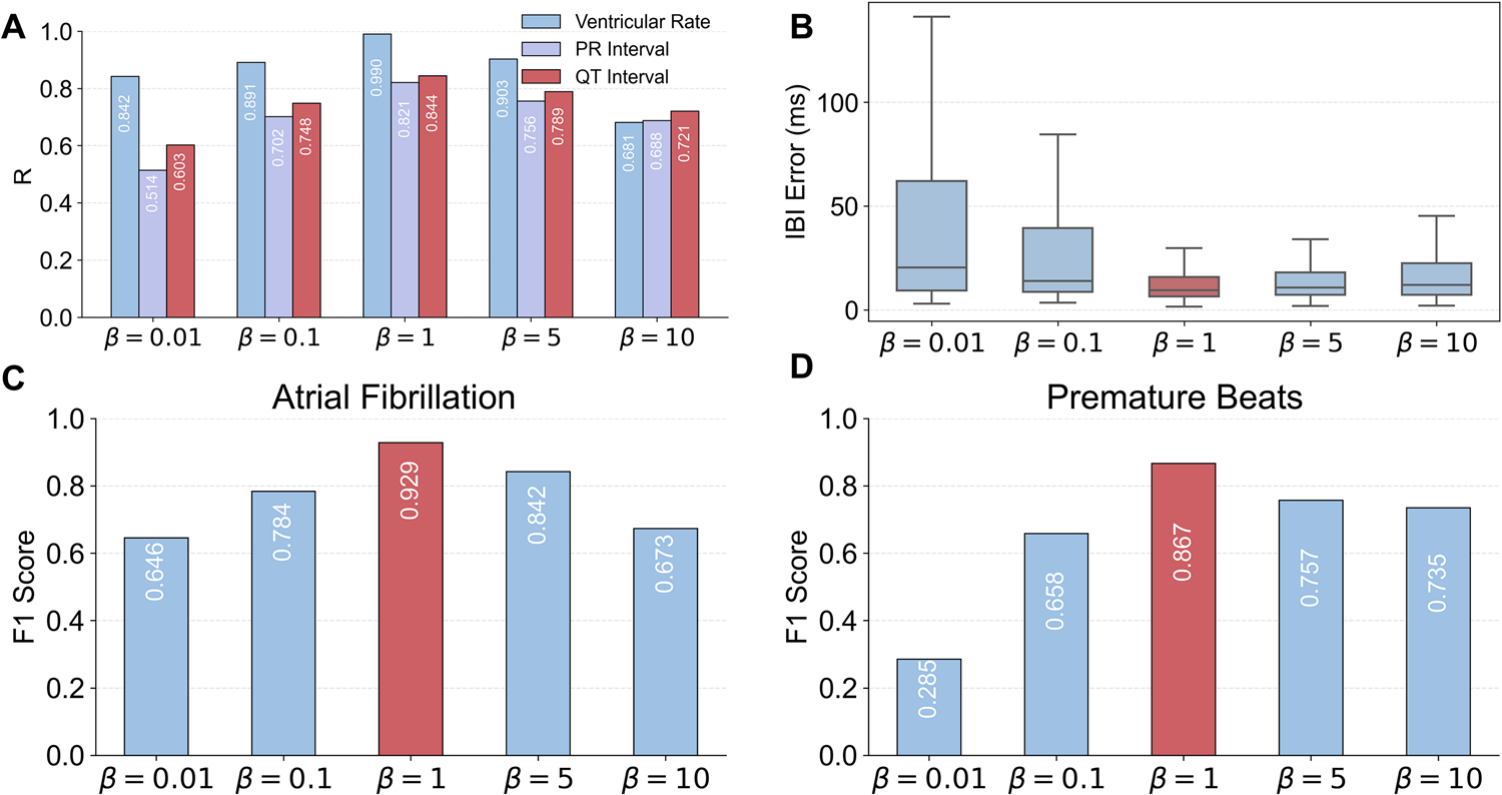
Impact of ECG semantic quality, controlled by the β value in the β‐VAE. (A) Impact of ECG semantic quality on factor‐to‐semantic readout performance. Numerical results of correlation (R) are provided in the plot (all p<0.001; n=9518). (B) Impact of ECG semantic quality on downstream HRV monitoring performance. The distribution of IBI errors is shown as box plots (all n=9518). (C) Impact of ECG semantic quality on downstream AF diagnosis performance (all n=9518). (D) Impact of ECG semantic quality on downstream PB diagnosis performance (all n=9518).

We next examine the sensitivity of the framework to the quality of the ECG semantic space. Our method follows prior work  [3, 5, 22, 33] and leverages a VAE formulation to disentangle ECG signals into a semantic reference space. In a VAE, the KL‐divergence (KLD) term regularizes the latent posterior toward a standard Gaussian prior and is widely recognized as key to affecting the quality of disentanglement in the learned representation. Therefore, we conduct an ablation study by varying the KLD weight β to control the quality of the ECG semantic space. Specifically, we evaluate β∈{0.01,0.1,1(paper setting),5,10}. As shown in Figure 7A, the performance of factor‐to‐semantic readouts peaks at β=1 and degrades when β becomes either smaller or larger. This trend is consistently observed in HRV monitoring (Figure 7B) as well as AF and PB diagnosis (Figure 7C,D). When β is small, the VAE approaches an autoencoder‐like framework, where latent factors tend to compress signal details rather than distangle structured cardiac semantics. When β is large, posterior collapse may occur, limiting the expressive capacity of the semantic space. Both extremes reduce the quality of the ECG semantic space. We also observe task‐dependent differences: when β is small, the model can still capture dominant global semantics, allowing ventricular rate estimation and AF detection, which rely on global rhythm characteristics, to remain relatively stable, whereas PB detection and IBI estimation, which depend on finer semantic structure, become more sensitive to semantic degradation. These results show that when the ECG semantic space becomes less structured and fails to disentangle meaningful cardiac dynamics, the cross‐modal alignment becomes less effective and downstream performance correspondingly degrades.

We then evaluate the proposed framework across multiple subpopulations, including age, gender, Body Mass Index (BMI), and pathology categories. The results indicate that our method does not exhibit statistically significant demographic bias within the studied cohort (detailed in Note S1.4).

To further evaluate generalization to new environments, we directly tested the pretrained model in four additional real‐life settings, including two inpatient hospital environments and two office environments (detailed in Note S1.5). In each environment, we collected data from 50 subjects under quasi‐static postures, including seated and supine conditions. For each subject, we recorded four 30‐s segments. The results showed that ventricular rate estimation remains largely consistent with the in‐domain outpatient dataset. PR and QT intervals show slight variations across environments, but the overall performance trends remain stable.

We finally evaluate the proposed method under different body movements of intensities (detailed in Note S1.6). Specifically, we consider three levels of body motion: (1) mild motion, where movement does not strongly disturb the thoracic region (simulated by recording subjects during desk work with light hand movements such as typing); (2) moderate motion, where movement affects the thoracic region (simulated by recording subjects in a standing condition with posture changes); and (3) high‐intensity motion, where movement causes substantial thoracic disturbance (simulated by recording subjects during walking). For each motion type, we recorded 20 subjects, and for each subject, we collected five 30‐s segments. The results demonstrate a clear trend. Under mild body motion, the performance remains close to the in‐domain quasi‐static setting, with a mean IBI error of 19.1 ms (95% CI: 18.4–19.8), compared with 19.0 ms (95% CI: 17.8–20.3) in the in‐domain quasi‐static condition. Under moderate motion, the performance shows a noticeable decline, with the mean IBI error increasing to 30.9 ms (95% CI: 27.2‐34.8). Under high‐intensity motion, the method is no longer able to provide reliable monitoring, with the mean IBI error further increasing to 214.3 ms (95% CI: 162.7–273.2). These results indicate that the proposed method cannot provide reliable monitoring during large body movements. However, we would also like to highlight that this does not invalidate its practical value for long‐term monitoring. In many realistic use scenarios, individuals still spend a substantial portion of time in low‐motion states, such as sleeping, resting, sitting, desk work, and other quasi‐static activities. When combined with body‐motion detection, the method remains applicable during these low‐motion intervals and can still provide meaningful monitoring coverage in practice.

## 4 Discussion

This study aimed to address the critical need for a new paradigm in contactless cardiac monitoring, moving beyond uninterpretable radio signals to establish a foundation for clinically semantic understanding. By bridging the semantic gap to radio signals, our framework not only achieves clinic‐level contactless cardiac monitoring with SOTA performance, but also transforms conventional black‐box radio cardiac sensing into a transparent and reliable solution for everyday cardiac health.

Central to the breakthroughs of this study is the development of a data‐driven semantic modeling framework. We formulate semantic learning as an information bottleneck problem and approximate its solution by exploiting intrinsic semantic invariances–both within the radio modality and across the radio–ECG domain. Built upon a multimodal deep learning architecture, our framework enforces intra‐modal compression to suppress non‐semantic variability and applies cross‐modal alignment with ECG to anchor the factor representation in physiological semantics. This framework factorizes high‐dimensional radio measurements into 64 interpretable semantic factors. Crucially, these semantic factors are not merely latent variables, they can be further visualized into both radio signal and the clinically familiar ECG waveform. This dual‐modality interpretability bridging low‐level signal dynamics and high‐level clinical semantic understanding, establishing a transparent and reliable foundation for downstream cardiac monitoring tasks.

Our findings are evaluated both quantitatively and qualitatively across multiple dimensions, ranging from the fidelity of the encoded semantics to their effectiveness in supporting clinical monitoring. Using a large‐scale cohort of 9518 outpatients, we demonstrated that the learned radio representations exhibit strong alignment with reference semantics. Cross‐modal analysis further revealed their physiological grounding, showing how latent semantic factors manifest coherently in both radio signals and clinically interpretable ECG waveforms. This semantic modeling leads to substantial gains in superior performance in cardiac monitoring tasks. For cardiac rhythm monitoring, our method significantly improves HRV accuracy, uncovering fine‐grained variations that traditional radio‐based methods fail to resolve. For arrhythmia diagnosis, our method not only improves performance in AF detection but enables robust identification of PB that remain indistinguishable under black‐box radio models. It achieves performance approaching ECG‐model‐based methods, while providing diagnostic reasoning that aligns with established clinical principles. Finally, we extend our approach to long‐term, real‐world deployment and demonstrate its robustness and efficiency in daily‐life monitoring scenarios.

While these results demonstrate the robustness and practical potential of the proposed framework, several aspects warrant further investigation. First, the current training requires synchronized radio–ECG pairs for cross‐modal anchoring, and temporal misalignment can degrade performance. Developing asynchronous or weakly supervised extensions to better leverage abundant ECG‐only and radio‐only data would improve scalability. Second, although the learned semantic factors exhibit strong statistical alignment with clinically meaningful semantics and support both prospective monitoring and retrospective interpretation, deeper cardiologist‐in‐the‐loop studies would further strengthen the assessment of clinical utility by delineating the method's practical boundaries and real‐world significance. Third, cross‐modal alignment depends on the quality of the constructed ECG semantic space. As shown in our sensitivity analysis, insufficient semantic disentanglement weakens downstream performance, suggesting that more advanced semantic modeling strategies may further enhance robustness. Also, the framework remains reliable under low body motion but degrades under high body motion. Although the method remains practically impactful for long‐term monitoring since low‐motion states constitute a large fraction of daily life, extending the method to better handle motion‐induced interference will broaden its applicability in real‐world scenarios. Finally, while contactless, zero‐operation monitoring offers clear convenience, it also raises privacy risks because physiological information may be captured without explicit user awareness or consent. Therefore, practical deployment should incorporate safeguards such as explicit user authorization and mechanisms to prevent unintended sensing  [43, 44]. Signal‐based identity verification may further help ensure authorized use  [45, 46].

In conclusion, our work establishes a new paradigm in contactless cardiac monitoring by demonstrating that radio signals can be semantically structured. This breakthrough enables clinically meaningful interpretation and delivers a substantial performance boost, approaching clinical‐grade reliability for radio‐based monitoring. Beyond the domain impact, it also exemplifies an emerging AI‐for‐Science pathway, where semantic structure is discovered directly from raw physical measurements rather than through empirical accumulation, paving the way toward data‐driven scientific discovery for emerging sensing modalities. As future work, we aim to extend this semantic framework to prospective validation across the continuum of clinical care, from early risk prediction to disease management, to demonstrate its real‐world impact on CVD health.

## 5 Methods

### 5.1 Contactless Radio Cardiac Measurement

Cardiac mechanical motions, though originating within the inner body, propagate through internal tissues and ultimately generate millimeter‐scale motions on the torso surface  [47, 48]. When radio signals reach the torso surface, reflections occur. These motions induce time‐varying changes in the propagation path length, which in turn linearly modulate the phase of the reflected signals by the following equation [49]:

(2) $$\begin{array}{c} \phi \left(t\right)=2\pi \frac{d(t)}{\lambda } \end{array}$$

where λ is the wavelength of radio signals, d(t) is the distance between the radio sensor and the position of reflection occurs, and t is sensing time. Therefore, the core idea of measuring cardiac motions using radio signals is to spatially sample reflections from the body surface and extract their phase variations. To implement this, we utilized a hardware platform from past work  [18], which features a 60GHz Frequency‐Modulated Continuous‐Wave radar with 4GHz bandwidth and a 2D antenna array, supporting full 3D spatial scanning around the torso. With a wavelength of 5mm, millimeter‐wave signals are highly sensitive to cardiac micro‐motions. In addition, they can penetrate clothing and blankets, enabling zero‐effort measurements without disrupting users' everyday life. The planar antenna array and wideband signals will be used by the beamforming to separate reflections coming from the different positions [49]:

(3) $$\begin{array}{c} x\left(p_{x,y,z},t\right)=\sum_{m=1}^{M}\sum_{n=1}^{N}e^{-j2\pi \frac{d_{m}\left(p_{x,y,z}\right)}{\lambda_{n}}}y_{m,n}\left(t\right) \end{array}$$

where $d_{m}\left(p_{x,y,z}\right)$ denotes the round‐trip distance through the voxel position $p_{x,y,z}$ in cartesian coordinates to the transmitter‐receiver antenna pair m, $x(p_{x,y,z},t)$ denotes the reflected signals from $p_{x,y,z}$ at time t, $\lambda_{n}$ is the wavelength corresponding to the frequency at the n‐th fast‐time sample within a chirp, $y_{m,n}\left(t\right)$ is the received signal at time t from the m‐th antenna pair and the n‐th fast‐time sample. After applying the beamforming, time‐domain received signals y(t) are separated into different reflected signals in voxels. These voxel signals are further processed into cardiac motion signals by following the signal processing introduced in past work  [50], which includes localizing torso surface reflections, extracting phase variations, and suppressing respiratory interference.

The final radio cardiac measurement is a 4D spatiotemporal tensor $X_{R}\in R^{X\times Y\times Z\times T}$ encoding cardiac motion across space (X,Y,Z) and time (T). This signal representation supports downstream semantic modeling and physiological interpretation. The detail of this signal processing is provided in Note S4.

### 5.2 Radio Semantic Representation Modeling Framework

Our framework adopts a multi‐modal deep learning architecture that leverages both intra‐modal and cross‐modal semantic invariance to approximate the objective function in Equation (1) and ultimately disentangle the signal into a meaningful semantic representation. The overall modeling pipeline consists of three key stages: ECG Semantic Space Pretraining, Radio Semantic Representation Learning, Test‐Time Semantic Projection. The corresponding methods are described below, with details provided in Note S5.

#### 5.2.1 ECG Semantic Space Pretraining

Our framework first leverages the ECG‐only dataset to construct a semantic reference space by learning to factorize ECG signals into semantic representations. Specifically, we implement a β‐VAE architecture comprising an ECG encoder $E_{E}\left(\cdot \right)$ and decoder $D_{E}\left(\cdot \right)$, both built as mirror‐symmetric 14‐layer 1D ResNet‐based networks [42, 51]. The model takes 10‐s, 12‐lead ECG segments $X_{E}$ as input, where the encoder extracts latent features $Z_{E}$, and the decoder reconstructs the original signal. Following past work  [52], the ECG waveforms are first quantized using μ‐law encoding to improve dynamic range handling. The model is trained using the β‐VAE objective, which combines a reconstruction loss and a Kullback–Leibler (KL) divergence term to encourage compact, disentangled representations. To further enhance semantic fidelity, we incorporated supervised regularization using disease labels available in the dataset. A linear classification head is attached to the latent space to perform multi‐label disease prediction, and the resulting cross‐entropy loss is incorporated into the total objective. The final loss function is formulated as:

(4) $$\begin{array}{cc} L_{\text{ECG}}= & \;E_{q(Z_{E}|X_{E})}\left[-\log p(X_{E}|Z_{E})\right]+\beta \;D_{\text{KL}}\left(q\left(Z_{E}|X_{E}\right)\;∥\;N\left(0,I\right)\right)\\ & +\lambda \;E_{q(Z_{E}|X_{E})}\left[-\log p(Y_{E}|Z_{E})\right] \end{array}$$

where $Y_{E}$ denotes the disease label, $q(Z_{E}|X_{E})$ is the variational posterior modeled by encoder, $p(X_{E}|Z_{E})$ is the reconstruction likelihood modeled by the decoder, and $p(Y|Z_{E})$ denotes the conditional probability distribution over disease labels parameterized by the probabilistic classifier. β and λ are hyperparameters that control the weight of the KL divergence and supervised loss, respectively. Once training is complete, the ECG encoder and decoder are frozen for use in the subsequent stage.

#### 5.2.2 Radio Semantic Representation Learning

Next, we leverage the Radio‐ECG paired dataset to learn the semantic representation of radio signals. This process includes: intra‐modal signal compression and cross‐modal semantic alignment. Specifically, we construct a radio encoder $E_{R}\left(\cdot \right)$ and decoder $D_{R}\left(\cdot \right)$ based on a hybrid ResNet architecture  [53] to jointly model the spatiotemporal structure of the input radio tensor $X_{R}$. In $E_{R}\left(\cdot \right)$, 3D convolutional layers first compress the input into a compact feature volume, after which a temporal encoder aggregates frame‐wise features to model longer‐range cardiac dynamics. Given a 10‐s radio segment, the encoder compresses the input into a compact latent representation $Z_{R}$, and the decoder reconstructs the original signal. To further enforce semantic invariance, we introduce a signal transformation T(·) that simulates realistic propagation‐induced variations in radio signals during contactless measurement. This transformation extracts non‐cardiac components from other recordings in the dataset and adds them into the target signal as semantic‐invariant perturbations. By regularizing the latent representations to remain consistent under such perturbations, we compel the model to compress propagation‐driven signal variations and instead focus on the underlying physiological semantics. We regularize the consistency by minimizing the MSE between the latent features of different transformed versions derived from the same input signal. For cross‐modal semantic alignment, we utilize synchronized ECG recordings. The frozen ECG encoder $E_{E}\left(\cdot \right)$ provides semantic references $Z_{E}$. We enforce alignment between $Z_{R}$ and $Z_{E}$ using a cosine similarity loss. We further regularize the alignment by reconstructing ECG waveforms from $Z_{R}$ using the frozen ECG decoder $D_{E}\left(\cdot \right)$, serving as an auxiliary semantic constraint. The final loss function is formulated as:

(5) $$\begin{array}{cc} L_{\text{Radio}}=\; & \underset{\text{Intra-modal semantic invariant compression loss}}{\underset{︸}{∥X_{R}-D_{R}\left(E_{R}\left(X_{R}\right)\right){∥}_{2}^{2}+\alpha \;{∥E_{R}\left(T\left(X_{R}\right)\right)-E_{R}\left(X_{R}\right)∥}_{2}^{2}}}\\ & +\underset{\text{Cross-modal semantic invariant alignment loss}}{\underset{︸}{\gamma \;\left(1-\cos\left(E_{R}\left(X_{R}\right),E_{E}\left(X_{E}\right)\right)\right)+\delta \;{∥X_{E}-D_{E}\left(E_{R}\left(X_{R}\right)\right)∥}_{2}^{2}}} \end{array}$$

where α, γ and δ are hyperparameters that balance the contributions of semantic invariance regularization and cross‐modal semantic alignment, respectively. In our implementation, we use α=0.1, γ=1, and δ=0.0001. We use above loss function serves as a practical, data‐driven approximation of the information bottleneck objective in Equation (1), guiding the model to learn compact and semantically meaningful radio representations. Once training is complete, the radio encoder and decoder are frozen for use during testing.

#### 5.2.3 Test‐Time Semantic Projection

During test‐time, only radio signals are required. The frozen radio encoder projects the incoming radio signal into the learned semantic space, enabling downstream monitoring and analysis. This semantic representation can be further decoded into both ECG and radio waveform domains via the frozen ECG and radio decoders, providing unified interpretability by bridging latent semantics and signal‐level expressions.

## 6 Experimental Section

### 6.1 Ethics Statement

This study was reviewed and approved by the Medical Research Institutional Review Board (IRB) of the First Affiliated Hospital of the University of Science and Technology of China under Application No. 2023KY056. All study subjects agreed to the use of their data for scientific research by signing the informed consent form and were informed of their right to withdraw their consent at any time. For the underage participants, informed consent was obtained from the legally authorized representatives. All participants volunteered for the project and did not receive any additional compensation.

### 6.2 Datasets

Our radio semantic representation modeling and evaluation are based on two primary data sources:

#### 6.2.1 Radio‐ECG Paired Dataset

We first construct a large‐scale radio‐ECG paired dataset comprising synchronized recordings of radio signals and 12‐lead ECG. A total of 9518 outpatient visitors were recruited during their routine ECG screenings, with radio signals collected simultaneously using our radio monitoring system. Each recording lasted between 10 to 30 s, with an average duration of 27 s. The cohort included individuals undergoing routine check‐ups as well as those seeking care due to discomfort. This clinically realistic distribution facilitates a representative evaluation of our method in practical clinical settings. All ECG recordings were annotated by certified cardiologists to provide diagnostic ground truth labels. Detailed procedures for data acquisition and dataset demographics are provided in Note S3.1.

#### 6.2.2 ECG‐Only Dataset

We construct a large‐scale ECG‐only dataset comprising ECG recordings and corresponding diagnostic labels from 85 669 subjects. This dataset was aggregated from five publicly available ECG databases: CPSC 2018  [54], PTB‐XL  [55], G12EL  [56], ECG‐Arrhythmia  [57], SPH  [58]. The duration of ECG recordings in these datasets ranges from 10 s to several hours, with an average of approximately 20 s per subject. Together, these datasets collectively comprise a large volume of ECG recordings, encompassing variations in age, gender, and clinical conditions. Dataset demographics are provided in Note S3.2.

### 6.3 Performance Evaluation Details

For the evaluation on Radio‐ECG paired datasets, we performed the K‐fold cross‐validation strategy (K = 7) to evaluate our model's performance. The dataset was randomly divided into seven equal‐sized subsets, ensuring that each fold contains non‐overlapping subjects. In each iteration, one fold is used as the test set, while the remaining folds are used for training and validation. This ensures strict subject separation between training and testing phases. The procedure is repeated seven times, and results are aggregated across folds to obtain overall performance metrics. Each input sample consists of a 10‐s segment, and test samples are generated every 5 s. For HRV evaluation, test samples from individual are concatenated to entire recording, based on which HRV metrics are computed. For arrhythmia diagnosis, performance is reported at the recording level, following the same evaluation protocol as in  [42].

For the evaluation on the case study of daily‐life monitoring, we directly deploy the trained model (from the above training stage) on full‐night radio recordings for evaluation. For HRV evaluation, which is clinically meaningful over extended durations, performance is assessed over the entire overnight recording. For arrhythmia diagnosis, a 5‐min sliding window is applied across the night. Within each window, predictions beyond 1.5×IQR above the 75th percentile are discarded as outliers to improve evaluation robustness. Diagnosis performance is then reported at the window level, using the same evaluation criteria as in the cross‐validation protocol.

### 6.4 Evaluation Metrics

We evaluate our framework across dimensions that reflect its clinical and representational effectiveness:

For HRV evaluation, all metrics are calculated based on beat‐to‐beat timing. The groundtruth beat (R peak) annotations are derived from synchronized ECG recordings using NeuroKit2  [59], an open‐source toolkit for ECG signal processing. This extraction protocol is also employed to obtain beat timings for our semantic ECG visualization results. Then we compute four standard HRV metrics to assess cardiac rhythm monitoring performance: **IBI** (inter‐beat interval), **RMSSD** (root mean square of successive IBI differences), which reflects short‐term heart rate variability; **SDNN** (standard deviation of normal‐to‐normal intervals), indicating the stability of autonomic regulation; and **pNN50** (percentage of successive IBIs differing by more than 50ms), which is closely related to parasympathetic activity. Performance is evaluated by measuring the absolute error between our predictions and the ground truth.

For semantic evaluation and arrhythmia diagnosis, groundtruth labels were annotated from the synchronized ECG recordings by two independent groups of certified cardiologists, who performed cross‐validation and mutual verification to ensure accuracy. The diagnostic performance is assessed using standard metrics, including **PRAUC** (Precision‐Recall Area Under the Curve), **Sensitivity** (true positive rate), **Specificity** (false alarm control), **F1 Score** (balance between precision and recall), and **Accuracy** (overall prediction correctness). Together, these metrics validate the semantic space's clinical reliability and interpretability.

## 7 Statistical Analysis

All statistical analyses were performed using Python with the SciPy and NumPy libraries. No additional transformation was applied to the primary semantic metrics (ventricular rate, PR interval, QT interval), as they are clinically defined quantities. Outliers were not removed unless explicitly stated; all reported statistics reflect the full evaluated cohort. Continuous variables are presented as mean ± standard deviation (SD) unless otherwise specified. For interbeat interval (IBI) error distributions, results are visualized using box plots showing median and interquartile range (IQR). 95% confidence intervals (CI) are obtained using 2000 bootstrap samples. The full outpatient cohort consisted of n=9518 subjects. All report sample sizes explicitly in the corresponding figure captions. Corresponding P‐values were calculated using two‐sided tests under the null hypothesis of zero correlation. Statistical significance was defined at α=0.001.

## Author Contributions

Y.C., J.C., T.Z. and C.G. conceived and designed the project. J.C. and X.C. contributed to the model design. J.C., H.W., G.X., Y.Y. and D.Z. designed and implemented the experiments to evaluate the performance. J.C. and H.W. analyzed the experimental results. J.C., Z.W. and D.Z. implemented the radio signal preprocessing and hardware system. J.C., Q.S. and Y.C. wrote the original manuscript. Y.C. supervised the work. All authors reviewed and approved the manuscript.

## Funding

This work was supported by the USTC Research Funds of the Double First‐Class Initiative YD2210002501.

## Conflicts of Interest

The authors declare no conflicts of interest.

## Supporting information


**Supporting File**: advs74810‐sup‐0001‐SupMat.pdf.

## Data Availability

The data collected or analyzed during this study are available under restricted access due to privacy regulations. Access requests can be directed to the corresponding author, with an expected response time of up to 60 days. Requesters should sign a data use agreement and submit a research proposal specifying the intended use. Access approval is contingent upon an ethical review to ensure compliance with institutional policies and legal requirements. Approved data use will be restricted to noncommercial research purposes.
