# Supplementary material for: Discovering Interpretable Semantics from Radio Signals for Contactless Cardiac Monitoring
Source: Adv Sci (Weinh). 2026 Mar 15;13(29):e24283. doi: 10.1002/advs.202524283 (PMC13205684; doi:10.1002/advs.202524283)
Supplement: Supplementary file 1 — Supporting File: advs74810‐sup‐0001‐SupMat.pdf. [file ADVS-13-e24283-s001.pdf]

Supplementary Information

Discovering Interpretable Semantics from Radio Signals for Contactless Cardiac Monitoring

Contents

1 Supplementary Note 1: Result Details **2**
- 1.1 Evolution of Semantic Factors Over Time 2
- 1.2 UMAP Projections of Dual-Modality Semantic Representations 3
- 1.3 Decision Analysis for Arrhythmia Classification 4
- 1.4 Demographic subgroup analysis 5
- 1.5 Generalization to unseen real-life environments 7
- 1.6 Impact of body motion interference 8

2 Supplementary Note 2: Experimental Details for Case Study **9**

3 Supplementary Note 3: Dataset Details **10**
- 3.1 Radio-ECG Paired Dataset 10
  - 3.1.1 Dataset Demographics 10
  - 3.1.2 Data Collection Details 10
- 3.2 ECG-Only Dataset 13

4 Supplementary Note 4: Contactless Radio Cardiac Measurement Details **14**

5 Supplementary Note 5: Radio Semantic Representation Modeling Framework **16**
- 5.1 ECG Semantic Space Pretraining 16
- 5.2 Radio Semantic Representation Learning 16
- 5.3 Test-Time Semantic Projection 19
- 5.4 Model Architecture 19

1 Supplementary Note 1: Result Details

1.1 Evolution of Semantic Factors Over Time

Complete understanding of cardiac status requires examining how these semantics evolve over time during continuous monitoring. Therefore, we demonstrate this dynamic property of semantic factors in long sequences. In Fig. S1a, we visualize a 10-second radio recording's semantic matrix, where each column represents a semantic factor and each row reflects the temporal progression over the recording. The matrix reveals that semantic representations vary dynamically over time, reflecting the evolving physiological context. To demonstrate the temporal consistency of our learned semantic space, we visualize the corresponding multi-modal waveforms (Fig.S1b) when specific semantic factors are manipulated. Specifically, we introduce a ”delay in atrial contraction” by increasing the latent factor variable correlated with the ventricular rate at a specific time segment. The resulting changes are consistently reflected in both the radio and ECG modalities, confirming that our semantic representations maintain temporal coherence and accurately reflect physiological variations.

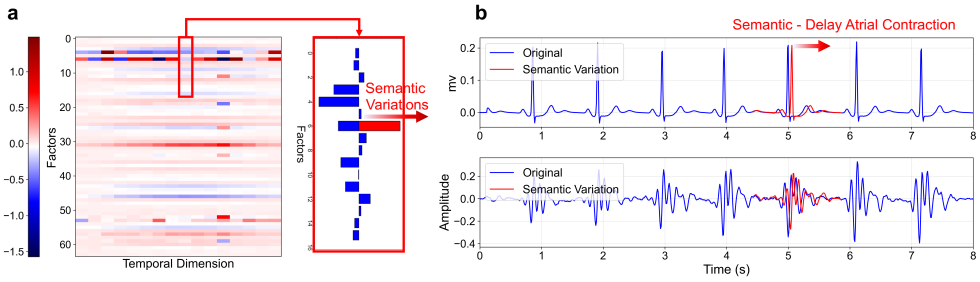

Fig. S1 Demonstration of semantic factor evolution over time. **a.** Semantic matrix of a 10-second radio recording, where each column represents a semantic factor and each row reflects its temporal progression. The right panel highlights a semantic variation simulating a “delay in atrial activation,” induced by increasing the value of the ventricular rate-related factor at a specific time segment. **b.** Multimodal waveform visualization reflecting changes driven by the semantic factor variation described above.

1.2 UMAP Projections of Dual-Modality Semantic Representations

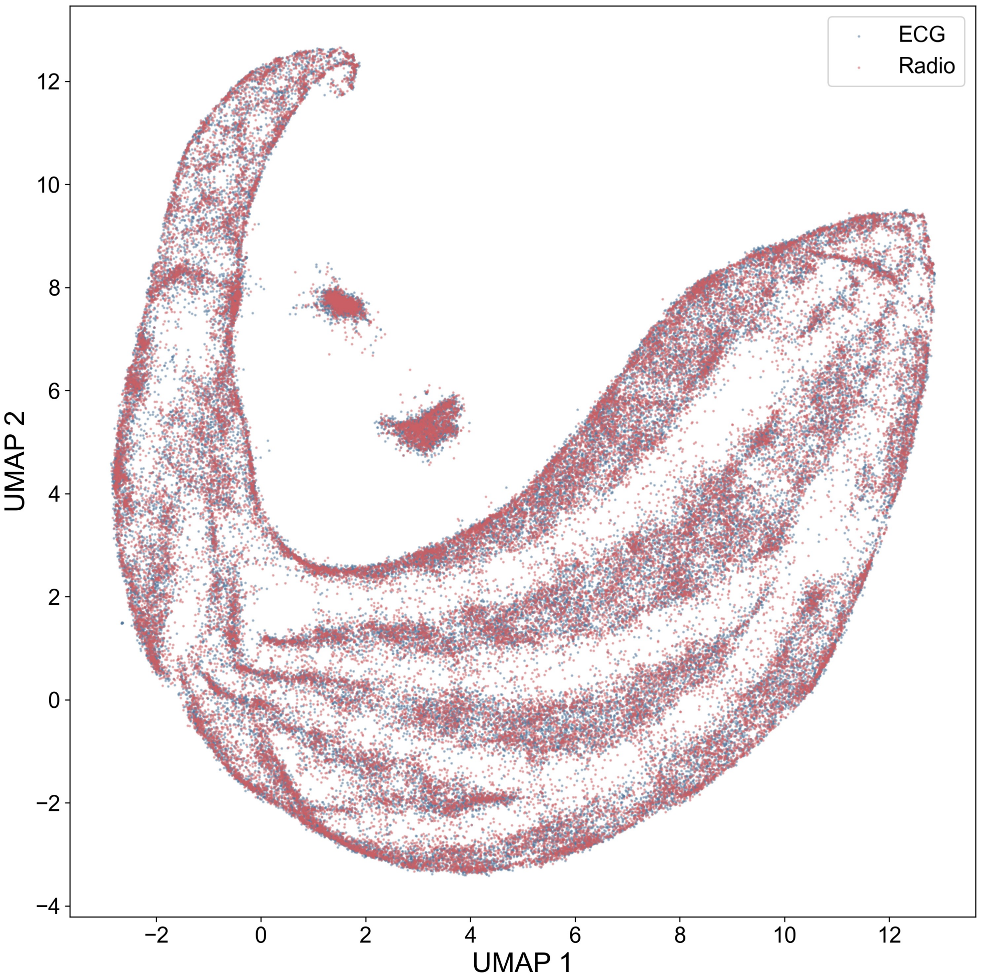

Fig. S2 UMAP projections of radio and synchronized-ECG semantic representations.

1.3 Decision Analysis for Arrhythmia Classification

To reveal how the model utilizes learned semantics for arrhythmia classification, we analyze its decision pathway as shown in Fig.S3. First, we apply Shapley Additive Explanations (SHAP) to the linear classifier built over the semantic representation to quantify the contribution of each factor. Next, the top two most important factors are varied from $-3\times$ to $+3\times$ their original values, and their semantic effects are examined via visualization. In the PB case, Factor 6 modulates the timing of an isolated R peak within consecutive beats, capturing the hallmark semantic of an ectopic beat with compensatory pause, while Factor 4 alters atrioventricular timing, corresponding to the clinical semantic of atrial/ventricular ectopy. In the AF case, the model emphasizes Factor 6 to encode beat-to-beat rhythm irregularity, and Factor 31 to represent the absence of P-wave activity—both hallmark criteria in clinical ECG-based AF diagnosis. These findings demonstrate that our semantic-based linear diagnostic pipeline follows the same decision logic used in clinical ECG interpretation, confirming that the model's predictions are driven by physiologically meaningful and clinically consistent semantics rather than black-box features.

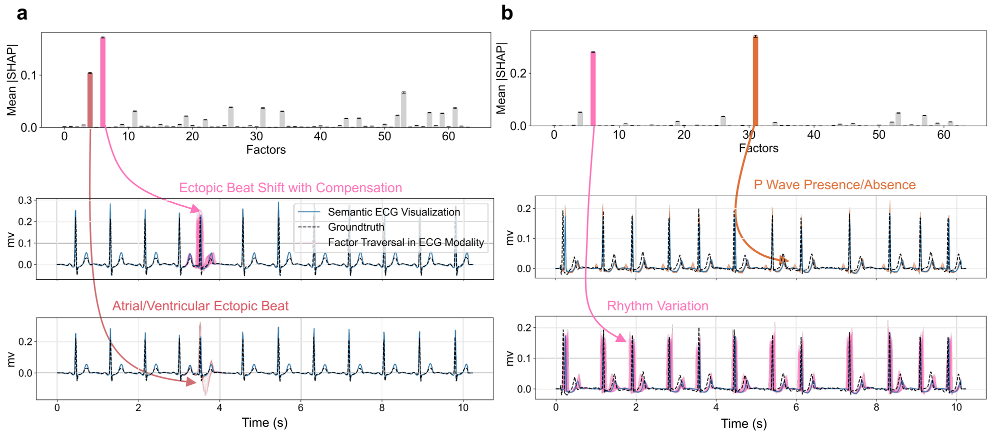

Fig. S3 Decision analysis of semantic factors for arrhythmia classification. **a.** PB case: the top row shows SHAP values for each semantic factor, indicating their contribution to the classifier's prediction. The two rows below visualize the semantic effects of the top two decision factors (Factors 6 and 4) in the ECG modality, corresponding to ectopic beat shift with compensation and atrial/ventricular ectopic beat semantics. **b.** AF case: the top row shows SHAP values, and the two rows below visualize the top two decision factors (Factors 31 and 6) in the ECG modality, corresponding to absence of P-wave semantics and irregular rhythm, respectively.

1.4 Demographic subgroup analysis

We validate the stability of the decoded semantic readouts and their alignment with clinically interpretable ECG semantic reference, including ventricular rate, PR interval, and QT interval. We first stratified the evaluation by gender. As shown in Fig. S4, performance remains consistent between male and female participants. We then stratified subjects into four age groups. As illustrated in Fig. S5, the proposed framework maintains relatively stable performance across age ranges. We observe a slightly higher correlation for the QT interval in the < 20 group, while the overall alignment remains consistent across groups. We further evaluated performance across BMI categories using Asian-specific criteria [1] by stratifying subjects into three groups: Others (BMI ≤ 23.0 kg/m^2), Overweight ($23.0 <$ BMI ≤ 27.5 kg/m^2), and Obesity (BMI > 27.5 kg/m^2). As shown in Fig. S6, results demonstrate consistent performance across BMI groups, suggesting robustness against variations in body composition that may affect radio signal propagation. Finally, we evaluated performance across pathology categories. To ensure statistical reliability, we grouped subjects into two clinically meaningful categories: ECG-normal and ECG-abnormal. The ECG-normal group includes recordings with sinus rhythm only, whereas the ECG-abnormal group includes recordings with any clinically identified ECG abnormalities. As shown in Fig. S7, our method maintains comparable performance across both groups, with small variations in PR interval and QT interval correlations.

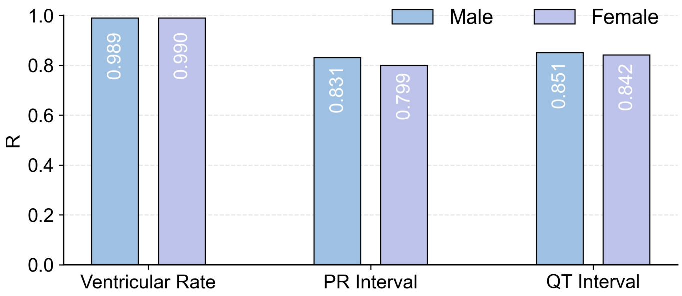

Fig. S4 Correlation between the decoded radio semantics and semantic groundtruth stratified by gender. Numerical results of correlation (R) are provided in the plot. (All $p < 0.001$; $n_{\text{male}} = 4179$, $n_{\text{female}} = 5339$).

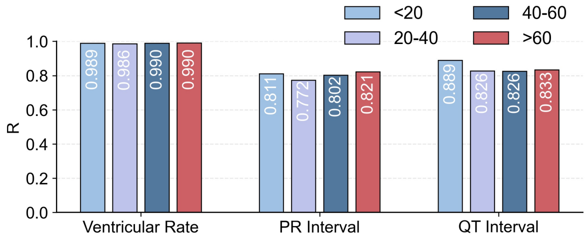

Fig. S5 Correlation between the decoded radio semantics and semantic groundtruth stratified by gender. Numerical results of correlation (R) are provided in the plot. (All $p < 0.001$; $n_{\text{male}} = 4179$, $n_{\text{female}} = 5339$).

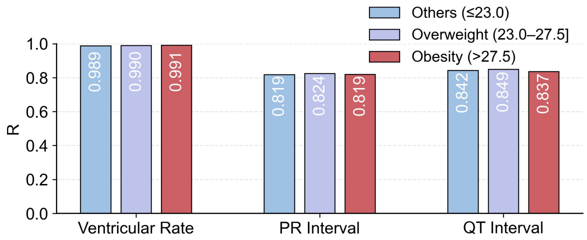

Fig. S6 Correlation between the decoded radio semantics and semantic groundtruth stratified by gender. Numerical results of correlation (R) are provided in the plot. (All $p < 0.001$; $n_{\text{male}} = 4179$, $n_{\text{female}} = 5339$).

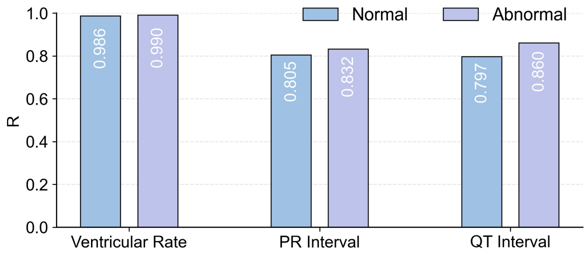

Fig. S7 Correlation between the decoded radio semantics and semantic groundtruth stratified by gender. Numerical results of correlation (R) are provided in the plot. (All $p < 0.001$; $n_{\text{male}} = 4179$, $n_{\text{female}} = 5339$).

1.5 Generalization to unseen real-life environments

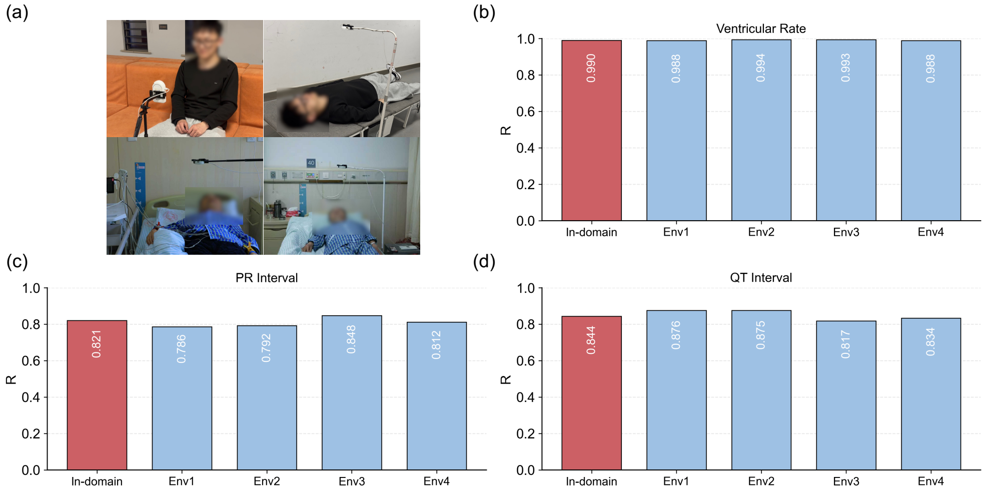

Fig. S8 Generalization to unseen real-life environments. (a) Demonstration of the four additional testing environments, including two inpatient hospital settings and two office settings. (b–d) Cross-environment evaluation results for ventricular rate, PR interval, and QT interval, respectively. (all $p < 0.001$; $n_{\text{in-domain}} = 9518$, $n_{\text{env1}} = n_{\text{env2}} = n_{\text{env3}} = n_{\text{env4}} = 200$)

1.6 Impact of body motion interference

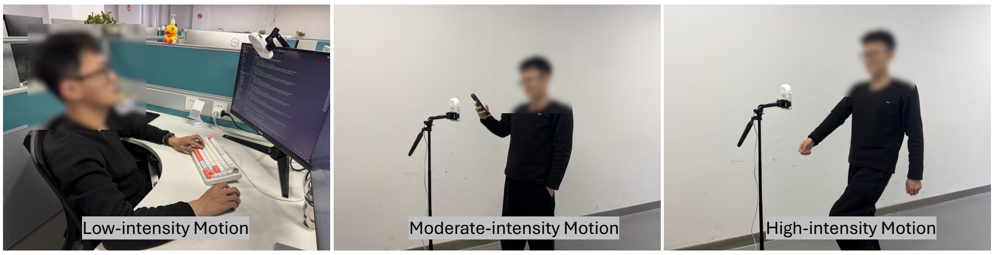

Fig. S9 Illustration of the three body-motion conditions considered in the additional experiments.

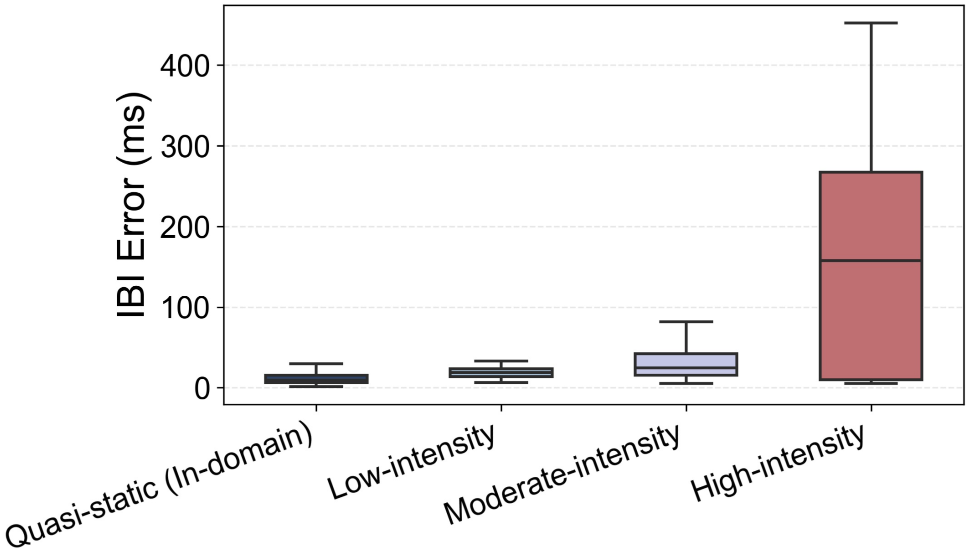

Fig. S10 Distribution of IBI error across quasi-static, low-intensity, moderate-intensity, and high-intensity body-motion conditions, shown as box plots. ($n_{\text{in-domain}} = 9518$, $n_{others} = 100$)

2 Supplementary Note 2: Experimental Details for Case Study

This case study directly evaluates the performance of the pretrained radio semantic model on overnight sleep monitoring data. The experiment involved 48 participants who reported discomfort and were scheduled for medical check-ups, comprising 20 males and 28 females. Before their hospital visits, we installed our radio monitoring device in each participant's bedroom and recorded overnight signals as they slept naturally. The radar device was mounted above the bed at a height of approximately 70cm. Simultaneously, ground-truth ECG signals were collected using a Holter monitor for validation purposes. Throughout the monitoring process, participants maintained their usual sleep routines without any intervention, aside from the presence of a radar sensor mounted above the bed. After the recordings, the ECG data were annotated by certified cardiologists to provide diagnostic reference for subsequent analysis. Based on these annotations, 6 participants were diagnosed with atrial fibrillation and 4 with premature beats.

The continuous overnight recordings are segmented using a sliding window approach, where each 5-second stride generates a 10-second input segment for evaluation. In contrast to the experimental tests conducted on outpatient cohorts where data acquisition is supervised by clinical staff to ensure a quasi-static supine posture, the overnight recordings inevitably include various body movements, such as turning over, getting in and out of bed, and postural adjustments. We provide a representative signal comparison in Fig. S11 to illustrate the impact of body motion. To mitigate the impact of such motion artifacts, we adopt the Zero Crossing Time (ZCT) method introduced in [2] to detect and exclude motion-contaminated segments. The remaining clean segments are subsequently used for downstream analysis and performance evaluation.

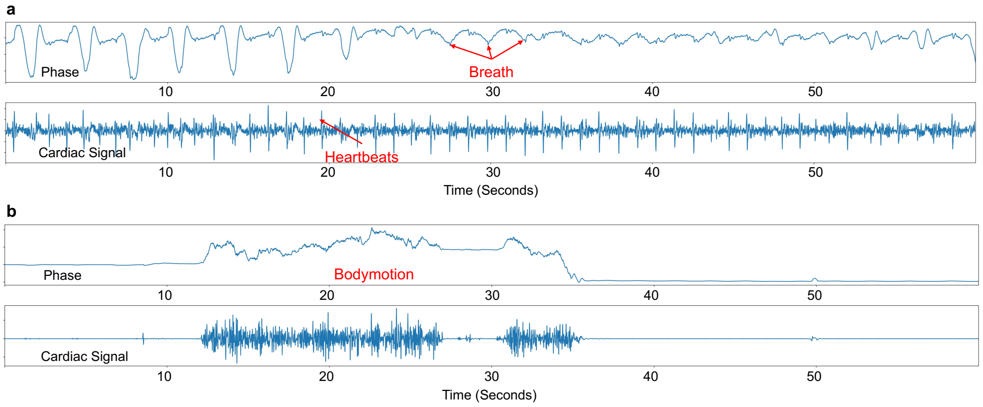

Fig. S11 Signal comparison between quasi-static and body motion conditions. (a) Example voxel signal showing phase variations (top) and corresponding cardiac signal (bottom) over one minute under quasi-static condition. **(b)** Example voxel signal showing phase variations (top) and corresponding cardiac signal (bottom) over one minute under body motion condition.

3 Supplementary Note 3: Dataset Details

3.1 Radio-ECG Paired Dataset

3.1.1 Dataset Demographics

The Radio-ECG Paired Dataset is a large-scale dataset comprising synchronized recordings of radio signals and 12-lead ECGs. Data collection was conducted as part of the outpatient service workflow at a leading hospital in Anhui Province, China. Participants included individuals undergoing routine physical examinations as well as those presenting with various symptoms or discomforts. In total, 9,518 outpatients were enrolled during their standard ECG screenings, with radio signals simultaneously acquired using our contactless radio monitoring system. This recruitment strategy ensures a clinically representative cohort, encompassing both ECG-normal individuals and patients with a diverse range of cardiovascular conditions. These include atrial fibrillation, premature beats, ST-T deviations, intraventricular blocks, atrioventricular blocks, axis deviations, and other complex physiological abnormalities. The condition distribution within the cohort is detailed in Table. S2. It should be noted that each outpatient may receive more than one cardiac condition diagnosis; therefore, the total count of cardiac condition types exceeds the total number of outpatients (9,518). Among them, 4,178 are male and 5,340 are female. The subjects' ages range from 15 to 97 years, and the age distribution is presented in Fig. S12. Each recording lasts between 10 and 30 seconds, with an average duration of 27 seconds. The distribution of recording durations per subject is illustrated in Fig.S13.

Table S1 Condition distribution in the Radio-ECG Paired Dataset

Condition	Number of Subjects
Normal	4786
Atrial Fibrillation	284
Premature Beat	592
ST&T Deviations	2797
Intraventricular Blocks	341
Atrioventricular Blocks	92
Axis Deviations	369
Others	893
**Total**	**9518**

3.1.2 Data Collection Details

In this study, we utilized a hardware platform based on our prior work [3] as the radio monitoring system. The system is implemented in a mobile phone–sized form, enabling seamless integration into daily life, as shown in Fig. S14. The hardware platform comprises two major components: a signal transceiver module and a data transmission module. The transceiver module is built upon a 60 GHz Frequency-Modulated Continuous-Wave (FMCW) radar chip (AWR6843AOP [4]) from Texas

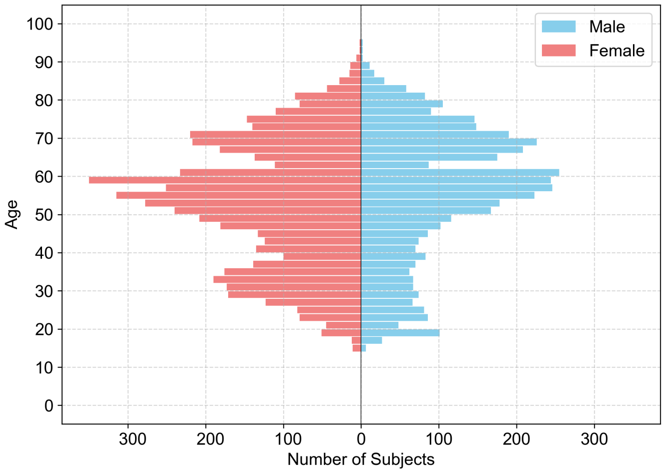

Fig. S12 Age distribution of the Radio-ECG Paired Dataset. Histogram of subject ages, separated by sex.

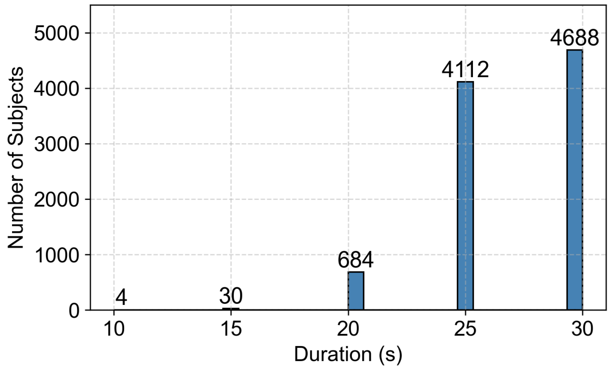

Fig. S13 Distribution of recording durations per subject.

Instruments. We activate 3 transmit (Tx) antennas and 4 receive (Rx) antennas to form a virtual 2D antenna array with 12 channels. A time-division multiplexing strategy is employed to ensure temporal orthogonality among the Tx antennas. During each sensing frame, the three Tx antennas sequentially emit chirps at 45ms intervals, allowing baseband signal acquisition from one Tx to all 4 Rx channels. Within each chirp, fast-time sampling is performed at a rate of 5 MHz with 256 samples per chirp capturing reflections across the entire swept bandwidth. The detailed radar chirp and frame configurations are provided in Table S2. With this setup, the radar achieves a frame rate of 100Hz. During continuous signal acquisition, the data is transmitted wirelessly to an online server via Wi-Fi communication for signal processing and storage.

As illustrated in Fig. S15, during data acquisition, participants were instructed to lie on a bed and remain in a quasi-static state. The radio monitoring device was positioned approximately 0.7 meters above the bed, resulting in a sensing distance

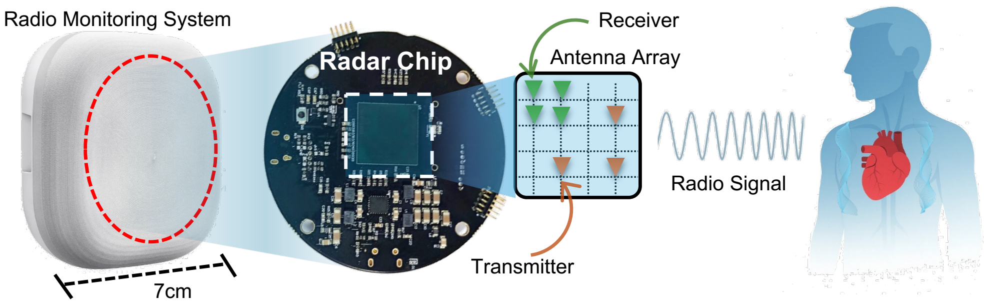

Fig. S14 Radio monitoring system used for contactless cardiac data acquisition.

Table S2 Radar configuration

Parameter	Value
Start frequency	60 GHz
Frequency slope	65 MHz/μs
Idle time	10 μs
Ramp end time	60 μs
Sample points	256
Sample rate	5 MHz
Frame periodicity	10 ms

of about 0.5 meters from the participant's chest, with slight variations depending on individual body size. Simultaneously, a synchronized 12-lead ECG was recorded. It is important to note that the paired ECG and radio measurements were used exclusively during the training phase of model development. In the test phase, only radio measurements are required, enabling a fully contactless monitoring approach.

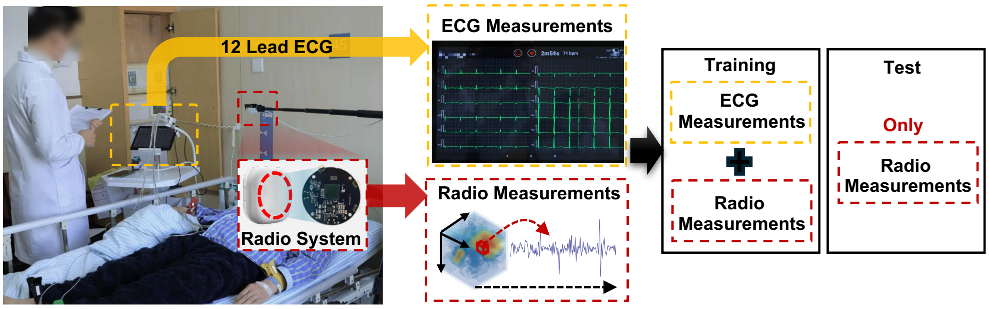

Fig. S15 Data collection setup.

3.2 ECG-Only Dataset

To construct the semantic space within the ECG domain, we curated a large-scale ECG-only dataset consisting of ECG recordings and corresponding diagnostic labels from 84,635 subjects. This dataset was aggregated from five publicly available ECG databases: CPSC 2018 [5], PTB-XL [6], G12EL [7], ECG-Arrhythmia [8], and SPH [9]. Together, these datasets offer extensive clinical coverage, including large-scale outpatient collections and multi-national cohorts, and also contain dedicated recordings for common cardiovascular conditions. This diversity ensures comprehensive representation across age, sex, and a wide spectrum of diagnostic categories, enabling the construction of a generalizable and semantically meaningful ECG representation space.

Given the variability in acquisition protocols across datasets, the quality of raw ECG signals is not uniform. In particular, common interferences such as power-line noise, baseline wander, and muscle artifacts due to poor electrode contact or body motion are frequently observed. To ensure consistent signal quality, we adopted a sequential noise reduction pipeline introduced in [8]. To further ensure the reliability of the training data, we followed the signal quality assessment strategy proposed in [9], employing the baseline Signal Quality Index (basSQI). For each 12-lead ECG recording, we computed basSQI individually for each lead and averaged the results. Records with average SQI below 0.7 were excluded to guarantee acceptable signal fidelity. The condition demographics of this ECG-only dataset are summarized in Table S3.

Table S3 Condition distribution in the ECG-Only Dataset

Condition	Number of Subjects
Atrial Fibrillation	11122
Premature Beat	6056
ST&T Deviations	24239
Intraventricular Blocks	10863
Atrioventricular Blocks	3278
Axis Deviations	6238
Others	12355
**Total**	**84635**

4 Supplementary Note 4: Contactless Radio Cardiac Measurement Details

Under the given radar configuration, the received signal for each frame can be expressed in complex matrix form as $Y_{M,N}(t)$, where M denotes the total number of transmitter–receiver antenna pairs, and N represents the number of fast-time sampling points per chirp. These raw radio reflections contain composite information from the surrounding environment, including static background, body movements, and subtle reflections from the torso. Our objective is to isolate the torso-reflected signals related to cardiac activity and extract the underlying cardio-motion dynamics. To achieve this, we implement a signal processing pipeline involving cardiac reflection extraction, motion interference suppression, and the final construction of the radio-based cardiac measurement, as shown in Fig. S16.

First, we apply a beamforming algorithm [10] to reconstruct spatial voxel signals from the raw received data. Specifically, the phase of each received signal is shifted according to the time delay corresponding to a specific 3D location (X, Y, Z), and signals are coherently accumulated across channels and chirp samplings. By leveraging the spatial diversity from the planar antenna array and wideband chirps, coherent integration enables the reconstruction of voxel reflections via:

$$x(x, y, z, t) = \sum_{m=1}^{M} \sum_{n=1}^{N} e^{-j2\pi \frac{d_m(x,y,z)}{\lambda_n}} \cdot y_{m,n}(t) \tag{1}$$

where $d_m(x, y, z)$ denotes the round-trip distance from the 3D Cartesian coordinate (x, y, z) to the m-th transmitter–receiver pair, and λ_n is the wavelength corresponding to the n-th fast-time sample.

Next, we localize the torso reflection by identifying voxels associated with respiratory motion. To achieve this, we follow the idea from [11] to compute the autocorrelation function (ACF) of each voxel's signal and locate the voxel exhibiting the highest ACF peak within a valid respiratory cycle. This voxel is selected as the center of torso reflection. Since cardiac mechanical motion propagates from within the body to the entire torso surface, we extract a 3D region of interest centered on the detected torso reflection, defined by a predefined bounding box that covers torso space.

Once the torso reflection is localized, we extract the motion component embedded within the voxel signal by computing the phase variation over time. However, in addition to cardiac motions, the torso surface also exhibits movements caused by respiration and voluntary or involuntary body motion. Considering their differing frequency distributions, we adopt a noise-robust differentiator to suppress low-frequency respiratory and motion-related interference while enhancing heart motion components. The second-order differentiator is defined as follows [12]:

$$s_0'' = \frac{(s_{-3} + s_3) + 2(s_{-2} + s_2) - (s_{-1} + s_1) - 4s_0}{16h^2} \tag{2}$$

where s_0'' refers to the second derivative at a particular time sample, s_i refers to the value of the time series i samples away, h is the frame periodicity between consecutive samples. Finally, the radio cardiac measurement is a 4D spatiotemporal tensor $X_R \in \mathbb{R}^{X\times Y\times Z\times T}$ encoding cardiac motion across space (X, Y, Z) and time (T).

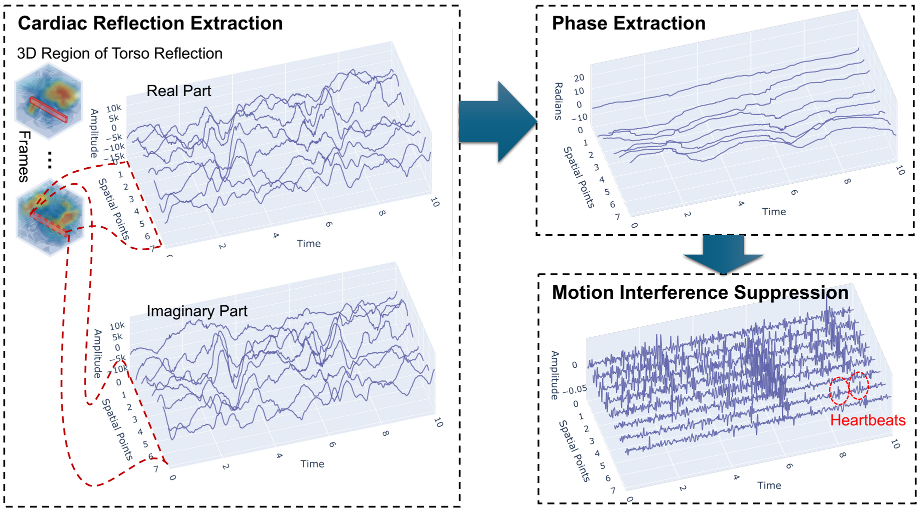

Fig. S16 Signal processing pipeline demonstration. The raw received signals are first processed via beamforming to reconstruct spatial voxel signals. The cardiac reflection signal, represented in complex form with real and imaginary components, is extracted from a 3D region of interest centered on the detected torso reflection. This region is defined by a predefined bounding box covering the entire torso space. The motion component is obtained by computing the temporal phase variation of the voxel signal. To suppress low-frequency respiratory and motion-related interference while enhancing cardiac dynamics, a noise-robust second-order differentiator is applied. Finally, the radio cardiac measurement is represented as a 4D spatiotemporal tensor $X_R \in \mathbb{R}^{X\times Y\times Z\times T}$.

5 Supplementary Note 5: Radio Semantic Representation Modeling Framework

As illustrated in Fig. S17, we design a multimodal deep learning framework to approximate the information bottleneck semantic modeling problem and to factorize the radio cardiac measurement into a structured semantic representation. This framework consists of three stages: ECG Semantic Space Pretraining, Radio Semantic Representation Learning, and Test-Time Semantic Projection.

5.1 ECG Semantic Space Pretraining

To establish a semantically meaningful latent space for ECG signals, we employ a β-VAE framework trained on our ECG-only dataset. The model consists of an encoder $E_E(\cdot)$ and decoder $D_E(\cdot)$, both designed to process 10-second segments of 12-lead ECG data. Prior to training, each ECG signal undergoes $\mu - law$ quantization [13] to enhance the encoding of fine-grained waveform details across varying amplitudes. The encoder maps the input X_E into a latent representation Z_E, which is then decoded back to reconstruct the original input. A linear classification head is further attached to the latent space to perform multi-label disease prediction. The overall training objective integrates three components: a reconstruction loss, a Kullback–Leibler (KL) divergence term, and a supervised cross-entropy loss based on disease labels as follow:

$$\begin{aligned}\mathcal{L}_{\mathrm{ECG}} = \mathbb{E}_{q(Z_E|X_E)}\left[-\log p(X_E|Z_E)\right] + \beta\, D_{\mathrm{KL}}\left(q(Z_E|X_E)\,\|\,\mathcal{N}(0,I)\right)\\ + \lambda\,\mathbb{E}_{q(Z_E|X_E)}\left[-\log p(Y_E|Z_E)\right]\end{aligned} \quad (3)$$

where Y_E denotes the disease label, $q(Z_E|X_E)$ is the variational posterior modeled by encoder, $p(X_E|Z_E)$ is the reconstruction likelihood modeled by the decoder, and $p(Y|Z_E)$ denotes the conditional probability distribution over disease labels parameterized by the probabilistic classifier, β and λ are hyperparameters that control the weight of the KL divergence and supervised loss, respectively. Once training is complete, the ECG encoder and decoder are frozen for use in the subsequent stage.

5.2 Radio Semantic Representation Learning

The semantic modeling can be formlaized as a classical information bottleneck problem [14, 15]: given radio measurements X_{R}, the objective is to learn latent representations Z_{R} that retain maximal mutual information with the desired cardiac semantics S, while minimizing mutual information with the radio signal itself X_{R}:

$$\min_{p(Z_{\mathrm{R}}|X_{\mathrm{R}})} I(Z_{\mathrm{R}}; X_{\mathrm{R}}) - \beta I(Z_{\mathrm{R}}; S) \quad (4)$$

where β is a Lagrange multiplier that controls the trade-off between compression and semantic preservation. In this work, we approximate this objective by combining intra-modal semantic-invariant compression and cross-modal semantic-invariant alignment. Specifically, we reduce redundancy in the radio signal through an intra-modal semantic-invariant compression strategy, thereby minimizing $I(Z_{\mathrm{R}}; X_{\mathrm{R}})$, and

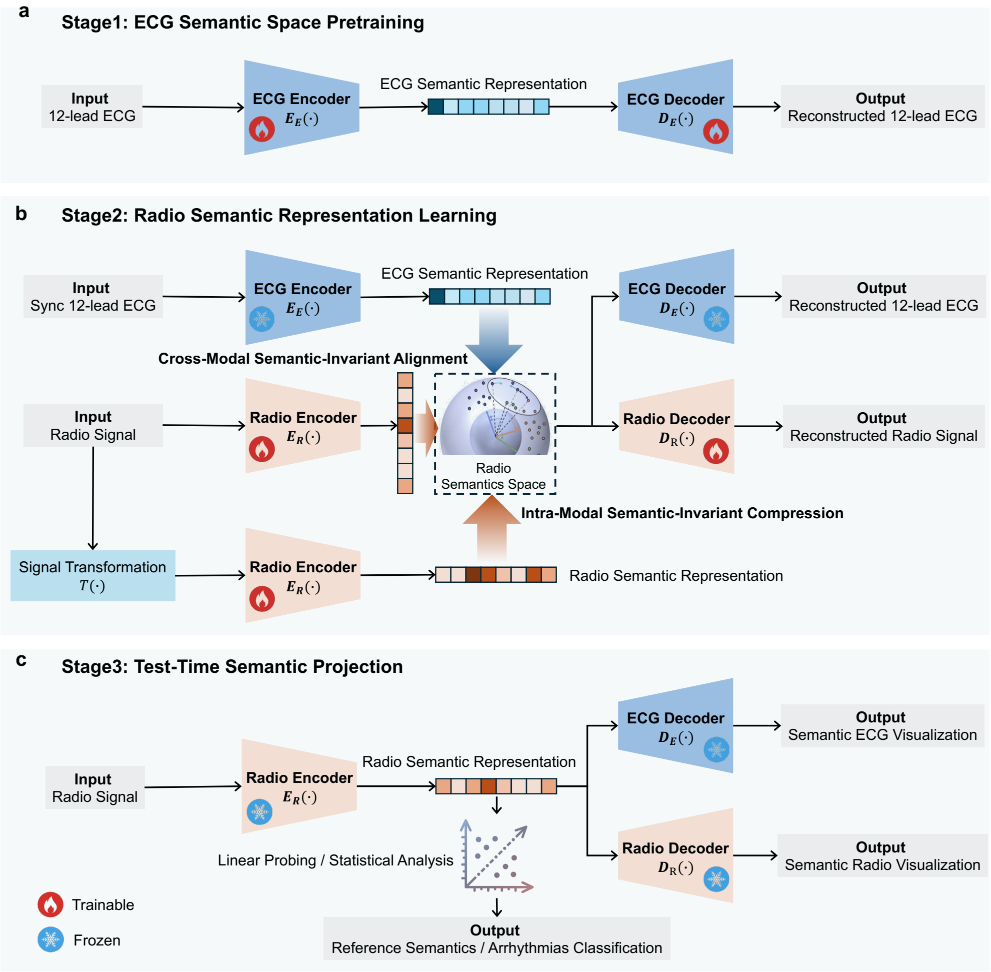

Fig. S17 Overview of the radio semantic representation modeling framework. **(a)** *ECG Semantic Space Pretraining:* A variational autoencoder is trained on a large-scale ECG-only dataset to learn a semantic space that captures meaningful cardiac dynamics. **(b)** *Radio Semantic Representation Learning:* By using the Radio-ECG paired dataset, we model the semantic representation of radio signals by combining intra-modal semantic-invariant compression and cross-modal semantic alignment. **(c)** *Test-Time Semantic Projection:* During inference, only radio signals are used. The learned encoder projects incoming signals into the semantic space, enabling downstream cardiac monitoring, diagnosis, and interpretable signal reconstruction.

simultaneously enforce semantic fidelity by aligning the learned radio representations with ECG-derived semantic references, effectively maximizing $I(Z_{\mathrm{R}}; S)$. This stage is trained on the Radio-ECG Paired dataset.

Intra-Modal Semantic-Invariant Compression: For radio cardiac measurements, voxel signals are inevitably mixed with reflections from surrounding

environmental objects. Under such conditions, the received signal can be modeled as:

$$y(t) = \sum_{k=1}^{K} \alpha_{p_k}(\lambda, t)\, \sigma_{p_k}(\lambda, t)\, e^{j\frac{4\pi}{\lambda} d_{p_k}(t)} \tag{5}$$

where K denotes the total number of effective propagation paths. The propagation characteristics of each path includes the amplitude attenuation $\alpha_{p_k}(\lambda, t)$, the radar cross section $\sigma_{p_k}(\lambda, t)$, and the propagation distance $d_{p_k}(t)$. Multiple factors influence these parameters in contactless measurements, such as the spatial layout and material properties of surrounding objects, and the physical characteristics of the human body (e.g., torso morphology, and surface reflectivity) [10]. Collectively, these factors introduce substantial non-cardiac signal variations into the received measurements. Although these variations typically change slowly over time and exhibit lower-frequency spectral patterns compared to cardiac activity, they often dominate the received signal compared to the millimeter-level cardiac motion. As a result, their presence inevitably interferes the radio measurement and direct compression tends to entangle irrelevant signal variations, thereby undermining semantic fidelity and degrading representation quality.

Fortunately, the interaction between these two components is inherently asymmetric: while dominant variations can affect the cardiac motion through leakage, the subtle cardiac motion does not, in turn, modulate the variation itself. This asymmetry enables us to effectively extract variation-only components. Leveraging the additive nature of multipath propagation in Equation 5, we can transform signals with a wide range of semantic-irrelavant variations by adding these components. These transformed signals are then used to enforce intra-modal semantic invariance during compression. This strategy turns the diversity of signal variations into a training advantage, enabling the model to filter out irrelevant variations while preserving the fidelity of cardiac semantics.

Specifically, the signal transformation $T(\cdot)$ is implemented as follows. Given an input raw IQ signal X_R, we randomly sample another signal $\tilde{X}_R$ from the training set and apply a low-pass filter $\mathcal{F}_{\text{LP}}(\cdot)$ with a cutoff frequency of 0.5 Hz to extract slow-varying, cardiac semantic-irrelevant variations outside the cardiac band:

$$V_{\text{non-cardiac}} = \mathcal{F}_{\text{LP}}(\tilde{X}_R).$$

We then inject this variation by directly adding it to the raw IQ signal,

$$X'_R = X_R + V_{\text{non-cardiac}},$$

and pass the composite signal through the standard signal processing pipeline to obtain the transformed view:

$$T(X_R) = \Phi(X'_R),$$

where $\Phi(\cdot)$ denotes the signal processing operations, including beamforming, phase extraction, and second-order differentiation (details are provided in Supplementary

Note 4). In this way, $T(\cdot)$ perturbs the input with semantic-irrelevant variations while preserving the underlying cardiac semantics.

Finally, the processing flow in this stage includes the standard compression of the input signal and the regularization of semantic invariance over the compressed features of transformed signals. The training objective is formulated as:

$$\mathcal{L}_{\text{Intra-Modal}} = \underbrace{\|X_R - D_R(E_R(X_R))\|_2^2}_{\text{Compression loss}} + \alpha \underbrace{\|E_R(T(X_R)) - E_R(X_R)\|_2^2}_{\text{Semantic invariance loss}} \tag{6}$$

Cross-Modal Semantic-Invariant Alignment: We propose to regulate the semantic modeling of radio signals by aligning the learned representations with the semantic reference from the ECG modality, thereby efficiently approximating $I(Z_{\text{R}}; S)$. Specifically, we freeze the pretrained ECG encoder $E_E(\cdot)$ and use it to provide semantic references $Z_E = E_E(X_E)$. To align the radio signal representation $Z_R = E_R(X_R)$ with the ECG semantics, we introduce a two-fold cross-modal alignment strategy: (1) we enforce representational similarity using a cosine similarity loss between Z_R and Z_E; (2) we project Z_R into ECG waveforms via the ECG frozen decoder $D_E(\cdot)$, using a reconstruction loss as an auxiliary semantic constraint. The final alignment loss is formulated as:

$$\mathcal{L}_{\text{Cross-Modal}} = \gamma \ \left(1 - \cos\left(E_R(X_R), E_E(X_E)\right)\right) + \delta \ \|X_E - D_E(E_R(X_R))\|_2^2 \tag{7}$$

The overall objective of this stage jointly optimizes both intra-modal and cross-modal constraints as:

$$\mathcal{L}_{\text{Radio}} = \mathcal{L}_{\text{Intra-Modal}} + \mathcal{L}_{\text{Cross-Modal}}. \tag{8}$$

5.3 Test-Time Semantic Projection

During test-time, only radio signals are required. The frozen radio encoder projects the incoming radio signal into the learned semantic space, enabling downstream monitoring and analysis. The resulting semantic representation can be used for linear probing or statistical analysis to uncover its relationship with cardiac semantics or clinical conditions, thereby supporting arrhythmia diagnosis. Furthermore, this semantic representation can be further decoded into both ECG and radio waveform domains via the frozen ECG and radio decoders, providing unified interpretability by bridging latent semantics and signal-level expressions.

5.4 Model Architecture

The radio semantics modeling framework includes pairwise radio encoder $E_R(.)$ and decoder $E_R(.)$ and pairwise Ecg encoder $E_E(.)$ and decoder $D_E(.)$.

ECG Model The ECG encoder $E_E(\cdot)$ and decoder $D_E(\cdot)$ are implemented as mirror-symmetric 12-layer networks based on a 1D ResNet architecture. The model takes 10-second, 12-lead ECG segments $X_E \in \mathbb{R}^{L \times T}$ as input, where $L = 12$ denotes

the number of leads and $T = 1024$ is the temporal length after zero-padding. Given a sampling rate of 100 Hz, the original 10-second ECG signal (1000 points) is zero-padded to 1024 for computational convenience. The encoder extracts latent representations Z_E, while the decoder reconstructs the original ECG waveform from the latent features. Each ResNet block consists of two convolutional layers with a kernel width of 15. The number of filters in each convolution is set to 32×2^k, where k is a stage index starting from 0 and incremented by 1 every fourth residual block. Temporal downsampling is applied every two residual blocks via strided convolution with a factor of 2. The decoder mirrors this structure, using transposed convolution to upsample the latent features back to the original temporal resolution.

Radio Model The radio cardiac measurements are represented as a four-dimensional spatiotemporal tensor: $X_R \in \mathbb{R}^{X \times Y \times Z \times T}$ where X, Y, and Z denote the spatial dimensions in Cartesian coordinates, and T denotes the temporal dimension. In our implementation, this tensor spans a physical field-of-view of 48 cm along the z-axis (depth), and 40 cm along both the x-axis (lateral) and y-axis (vertical), over a 10-second duration. This spatiotemporal space is discretized into a grid of size $8 \times 8 \times 8 \times 1024$. However, directly modeling such a high-dimensional 4D tensor imposes significant computational and memory burdens.

To enable tractable yet expressive modeling, we adopt a dimensionality reduction strategy inspired by prior work [16], which demonstrated that projecting the 4D tensor into lower-dimensional views can effectively approximate full 4D modeling performance. Specifically, we derive two three-dimensional representations by marginalizing over one spatial dimension each. The first view $X_{R1} \in \mathbb{R}^{Y \times Z \times T}$ is obtained by averaging over the x-axis, thereby preserving the depth–height–time (z–y–t) structure. The second view $X_{R2} \in \mathbb{R}^{X \times Z \times T}$ is obtained by averaging over the y-axis, thereby preserving the depth–width–time (z–x–t) structure. As illustrated in Fig. S18a, the model processes both X_{R1} and X_{R2} to extract semantic representations from the projected radio signal.

As shown in Fig. S18a, we design a hybrid ResNet-based encoder architecture to model the spatiotemporal structures of the two projected 3D radio tensors $X_{R1} \in \mathbb{R}^{Y \times Z \times T}$ and $X_{R2} \in \mathbb{R}^{X \times Z \times T}$. Following the strategy in [17], we address the inherent scale mismatch between the temporal and spatial dimensions (the temporal axis being significantly longer than the spatial axes) by adopting a two-stage feature extraction process. In the first stage, we apply a spatial–temporal encoder to each input tensor in parallel. Each branch jointly extract local spatiotemporal features across the two spatial dimensions and time. This stage progressively compresses the spatial dimensions while preserving the full temporal resolution. The resulting features from both branches are then concatenated along the channel dimension, forming a unified spatiotemporal representation. In the second stage, we use a temporal encoder to further process this merged feature map along the temporal axis, enabling hierarchical abstraction of temporal dynamics. The final output of the encoder is the latent semantic representation Z_R, which serves as the input to subsequent compression, alignment, and decoding modules.

The spatial–temporal encoder begins with a 3D convolutional layer, followed by a sequence of hybrid 3D ResNet blocks and Down3D modules that progressively extract

spatial features and reduce spatial resolution. Each hybrid 3D ResNet block, as illustrated in Fig. S18b, comprises multiple layers of GroupNorm, nonlinearity activation, and Conv3D pairs with varied temporal kernel sizes. Specifically, we employ two types of 3D convolutions: a $(1, 3, 3)$ kernel that captures spatial structure within individual time frames, and a $(3, 3, 3)$ kernel that jointly models short-term temporal–spatial dynamics. These complementary convolutions are designed to efficiently compress spatial information while preserving essential temporal dependencies, facilitating effective feature extraction across the spatiotemporal domain.

For the temporal encoder, we adopt the same architectural design as used in the ECG model, using a 12-layer 1D ResNet backbone. This encoder processes the temporally preserved features from the spatial–temporal encoder and progressively refines them along the time dimension. Through hierarchical temporal feature extraction, it compresses the representations into the final latent semantic representation Z_R.

The decoder networks for radio signals also adopt mirror-symmetric architectures relative to their corresponding encoders, ensuring architectural consistency and effective signal reconstruction.

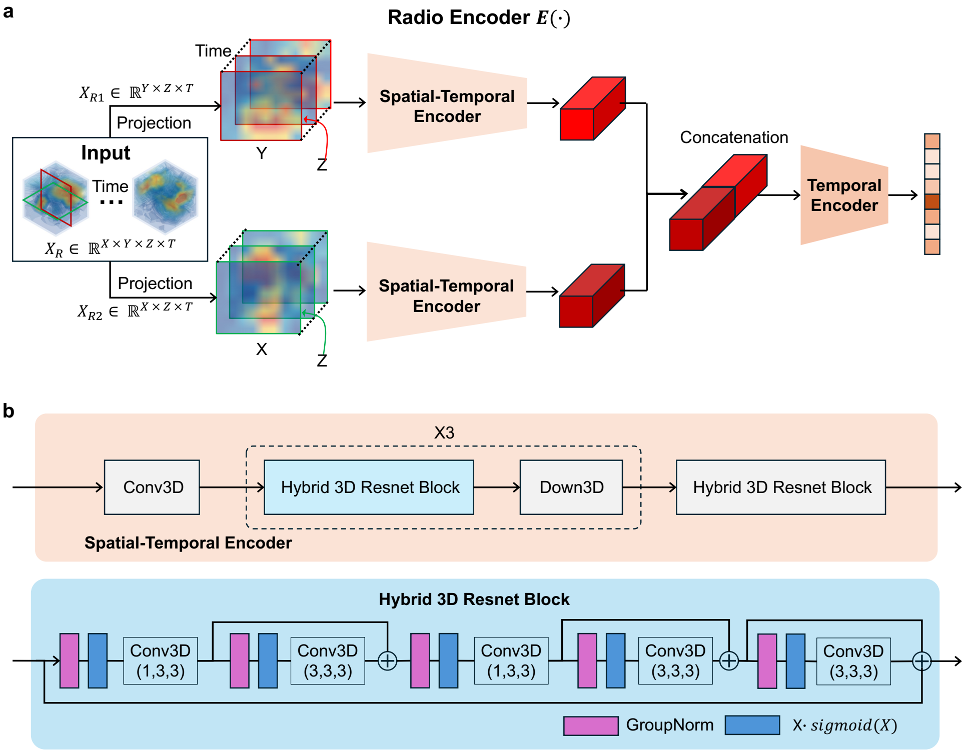

Fig. S18 Model architecture of the radio signal encoder. (a) Overview of the hybrid encoder design. The 4D spatiotemporal radio tensor is projected into two complementary 3D projections $X_{R1} \in \mathbb{R}^{Y\times Z\times T}$ and $X_{R2} \in \mathbb{R}^{X\times Z\times T}$, which are processed by parallel spatial–temporal encoders. Features are concatenated and further modeled by a temporal encoder to produce the semantic representation Z_R. **(b)** Structure of a hybrid 3D ResNet block used in the spatial–temporal encoder. It includes GroupNorm, nonlinearity activation, and pairs of Conv3D layers with $(1, 3, 3)$ and $(3, 3, 3)$ kernels to capture local and short-term spatiotemporal patterns.

References

[1] K. Tan *et al.*, "Appropriate body-mass index for asian populations and its implications for policy and intervention strategies," *The lancet*, 2004.

[2] S. Dong, L. Wen, Y. Li, J. Lu, Z. Zhang, C. Yuan, and C. Gu, "Remote respiratory variables tracking with biomedical radar-based iot system during sleep," *IEEE Internet of Things Journal*, vol. 11, no. 11, pp. 19 937–19 948, 2024.

[3] Y. Yuan, J. Chen, D. Zhang, R. Geng, H. Gong, G. Xu, Y. Pu, Z. Lu, Y. Hu, D. Zhang *et al.*, "Atrial fibrillation detection via contactless radio monitoring and knowledge transfer," *Nature Communications*, vol. 16, no. 1, p. 4317, 2025.

[4] Texas Instruments, "AWR6843AOP: Single-Chip 60 to 64GHz mmWave Sensor with Antennas-On-Package (AOP)," https://www.ti.com/product/AWR6843AOP, 2024, accessed: Sep. 18, 2025.

[5] F. Liu, C. Liu, L. Zhao, X. Zhang, X. Wu, X. Xu, Y. Liu, C. Ma, S. Wei, Z. He *et al.*, "An open access database for evaluating the algorithms of electrocardiogram rhythm and morphology abnormality detection," *Journal of Medical Imaging and Health Informatics*, vol. 8, no. 7, pp. 1368–1373, 2018.

[6] P. Wagner, N. Strodthoff, R.-D. Bousseljot, D. Kreiseler, F. I. Lunze, W. Samek, and T. Schaeffter, "Ptb-xl, a large publicly available electrocardiography dataset," *Scientific data*, vol. 7, no. 1, pp. 1–15, 2020.

[7] E. A. P. Alday, A. Gu, A. J. Shah, C. Robichaux, A.-K. I. Wong, C. Liu, F. Liu, A. B. Rad, A. Elola, S. Seyedi *et al.*, "Classification of 12-lead ecgs: the physionet/computing in cardiology challenge 2020," *Physiological measurement*, vol. 41, no. 12, p. 124003, 2020.

[8] J. Zheng, H. Guo, and H. Chu, "A large scale 12-lead electrocardiogram database for arrhythmia study (version 1.0. 0)," *PhysioNet 2022Available online: http://physionet. org/content/ecg-arrhythmia/1.0. 0/(accessed on 23 November 2022)*, 2022.

[9] H. Liu, D. Chen, D. Chen, X. Zhang, H. Li, L. Bian, M. Shu, and Y. Wang, "A large-scale multi-label 12-lead electrocardiogram database with standardized diagnostic statements," *Scientific data*, vol. 9, no. 1, p. 272, 2022.

[10] M. A. Richards *et al.*, *Fundamentals of radar signal processing.* Mcgraw-hill New York, 2005.

[11] F. Wang, F. Zhang, C. Wu, B. Wang, and K. R. Liu, "Vimo: Multiperson vital sign monitoring using commodity millimeter-wave radio," *IEEE Internet of Things Journal*, vol. 8, no. 3, pp. 1294–1307, 2020.

[12] P. Holoborodko, "Noise robust differentiators for second derivative estimation," http://www.holoborodko.com/pavel/downloads/NoiseRobustSecondDerivative, 2014, accessed: Aug. 4, 2025.

[13] A. Van Den Oord, S. Dieleman, H. Zen, K. Simonyan, O. Vinyals, A. Graves, N. Kalchbrenner, A. Senior, K. Kavukcuoglu *et al.*, "Wavenet: A generative model for raw audio," *arXiv preprint arXiv:1609.03499*, vol. 12, p. 1, 2016.

[14] D. Gündüz, Z. Qin, I. E. Aguerri, H. S. Dhillon, Z. Yang, A. Yener, K. K. Wong, and C.-B. Chae, "Beyond transmitting bits: Context, semantics, and task-oriented communications," *IEEE Journal on Selected Areas in Communications*, vol. 41, no. 1, pp. 5–41, 2022.

[15] S. Barbarossa, D. Comminiello, E. Grassucci, F. Pezone, S. Sardellitti, and P. Di Lorenzo, “Semantic communications based on adaptive generative models and information bottleneck,” *IEEE Communications Magazine*, vol. 61, no. 11, pp. 36–41, 2023.

[16] M. Zhao, Y. Tian, H. Zhao, M. A. Alsheikh, T. Li, R. Hristov, Z. Kabelac, D. Katabi, and A. Torralba, “Rf-based 3d skeletons,” in *Proceedings of the 2018 Conference of the ACM Special Interest Group on Data Communication*, 2018, pp. 267–281.

[17] Y. Xing, Y. Fei, Y. He, J. Chen, J. Xie, X. Chi, and Q. Chen, “Large motion video autoencoding with cross-modal video vae,” *arXiv preprint arXiv:2412.17805*, 2024.
